# Therapy-induced cholesterol biosynthesis drives lung cancer dormancy and drug resistance

**DOI:** 10.1172/JCI191735

**Published:** 2026-04-15

**Authors:** Yikai Zhao, Yijia Zhou, Linnuo Pan, Geng G. Tian, Hsin-Yi Huang, Shijie Tang, Ming Lu, Zhangsen Zhou, Peng Zhang, Luonan Chen, Lele Zhang, Liang Hu, Hongbin Ji

**Affiliations:** 1 Key Laboratory of Multi-Cell Systems, Shanghai Institute of Biochemistry and Cell Biology, Center for Excellence in Molecular Cell Science, Chinese Academy of Sciences, Shanghai, China.; 2 School of Life Science and Technology, Shanghai Tech University, Shanghai, China.; 3 School of Agriculture and Biology, Shanghai Jiao Tong University, Shanghai, China.; 4 Key Laboratory of Nutrition, Metabolism and Food Safety, Shanghai Institute of Nutrition and Health, University of Chinese Academy of Sciences, Chinese Academy of Sciences, Shanghai, China.; 5 Department of Thoracic Surgery, Shanghai Pulmonary Hospital, School of Medicine, Tongji University.; 6 School of Life Science, Hangzhou Institute for Advanced Study, University of Chinese Academy of Sciences, Hangzhou, China.; 7 School of Mathematical Sciences and School of Artificial Intelligence,; 8 Central Laboratory, Innovation and Incubation Center, Shanghai Pulmonary Hospital, School of Medicine, Tongji University.; 9 Shanghai Institute of Thoracic Oncology, Shanghai Chest Hospital, and; 10 Shanghai Key Laboratory of Thoracic Tumor Biotherapy, Shanghai Chest Hospital, Shanghai Jiao Tong University School of Medicine, Shanghai, China.

**Keywords:** Cell biology, Metabolism, Cancer, Drug therapy, Lung cancer

## Abstract

Complete response is rarely observed in lung cancer molecular targeted therapy, despite great clinical success. Here, we found that molecular therapy targeted toward EGFR mutant, KRAS mutant, or ALK fusion lung cancer induced cholesterol biosynthesis, which promoted cancer cells to enter dormancy and thus escape drug killing. Combined statin treatments effectively blocked cholesterol biosynthesis, prevented cancer cells from entering dormancy, and thus resulted in dramatic tumor regression. We further identified a subpopulation of cycling cancer cells that persisted during molecular targeted therapy and remained sensitive to aurora kinase inhibitors. Triple-targeting cholesterol biosynthesis, aurora kinase, and individual oncogenic drivers almost eradicated all the cancer cells. Therapy-induced cancer dormancy was mainly attributed to activation of unfolded protein response, specifically the PERK-eIF2α axis, which triggers cholesterol biosynthesis and AKT signaling. Collectively, this work uncovers an unexpected role of a therapy-induced prosurvival program in promoting cancer dormancy and provides a potentially effective strategy to prevent drug resistance.

## Abstract 2

Cholesterol synthesis drives cancer dormancy and drug resistance

## Graphical abstract

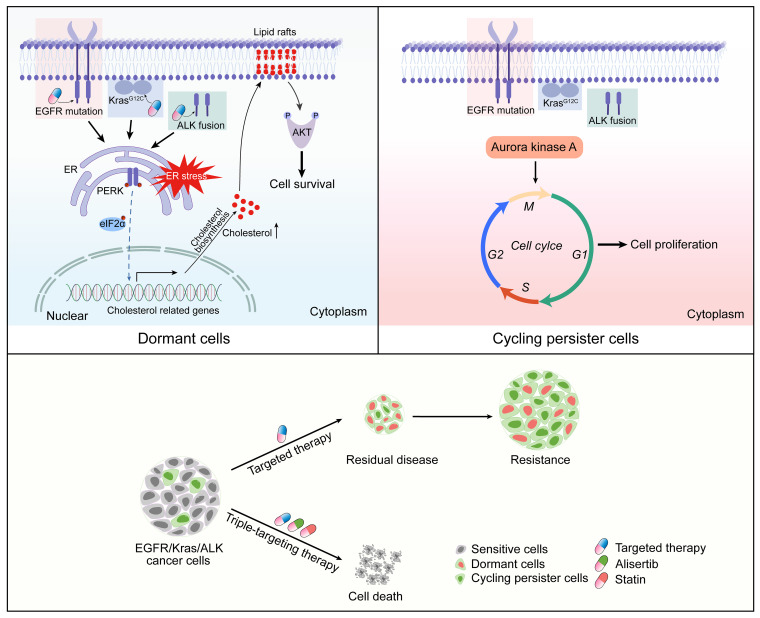

## Introduction

EGFR and KRAS mutations and ALK rearrangements are among the most prevalent oncogenic drivers in lung adenocarcinoma (LUAD) (1–3). Therapeutic targeting of these drivers has made significant progress and gained impressive clinical benefits (4, 5). For example, tyrosine kinase inhibitors (TKIs) such as gefitinib and osimertinib work effectively for patients with EGFR-mutant LUAD, whereas alectinib or lorlatinib work well for patients with ALK-fusion LUAD, and sotorasib and adagrasib, 2 specific inhibitors of KRAS G12C mutation, are frequently administered to patients with KRAS^G12C^ LUAD (5). Despite these encouraging observations, molecular targeted therapy frequently encounters an incomplete response, and patients’ disease relapses almost inevitably (6). Cancer dormancy, referring to the state in which cancer cells maintain survival but not rapid proliferation, remains a huge barrier for effective molecular targeted therapy in lung cancer (7).

A good example of cancer dormancy in the clinic is minimal residual disease, in which a small population of malignant cells persists during targeted therapy, often evading eradication. The downregulation of cyclin D1 and the upregulation of cyclin-dependent kinase inhibitor p27 frequently are apparent in dormant cells (8), whose positivity serves as the indication of dormancy (9). On 1 hand, these dormant cancer cells temporarily halt cell division and even enter a G0 phase, enabling them to evade the cytotoxic effects of anticancer drugs (10–12). On the other hand, these cells can reenter the cell cycle and resume growth once they gain sufficient strength genetically or epigenetically, or the microenvironment becomes more favorable. How cancer dormancy is triggered and maintained during molecular targeted therapy remains an important question for the development of better strategies to overcome dormancy-related drug resistance.

Cholesterol is synthesized through the mevalonate (MVA) pathway via a complex process involving the sequential production of HMG-CoA, MVA, and squalene (13). Cholesterol is an essential component of cell membranes and serves as a precursor for steroid synthesis. It plays an important role in the formation of lipid rafts—a specialized membrane microdomain gathering various signaling molecules. These lipid rafts facilitate the firing of prosurvival signaling and thus contribute to the development of drug resistance. For example, the activation of AKT signaling, downstream of multiple RTKs preferentially located at lipid rafts, promotes cancer cell survival, which is presumably important for dormancy maintenance.

Through integrative analyses of mouse models and clinical data, we identify here a therapy-induced prosurvival program: molecular targeted therapy activates cholesterol biosynthesis, which contributes to cancer dormancy via promoting cell survival, eventually leading to drug resistance. Based on mechanistic study and the additional finding of another cycling cancer cell subpopulation, we propose a triple-targeting strategy proven to be highly effective in overcoming drug resistance associated with cancer dormancy.

## Results

### Targeted therapy activates cholesterol biosynthesis.

To investigate cancer dormancy and drug-resistance acquisition following molecular targeted therapy, we first established an EGFR-mutant lung cancer (PC9 cells) xenograft mouse model and treated these tumors with TKI (Supplemental Figure 1, A and B; supplemental material available online with this article; https://doi.org/10.1172/JCI191735DS1). The tumors shrank dramatically after 1 week of gefitinib treatment. However, minimal residual tumors persisted and regrew after approximately 50 days of TKI treatments (Supplemental Figure 1B). We did not detect T790M mutation in these tumors (Supplemental Figure 1C), which accounts for approximately 50% to approximately 60% of acquired resistance to first- and second-generation EGFR TKI (14). This model recapitulates the clinical observation of cancer dormancy and drug resistance.

**Figure 1:**
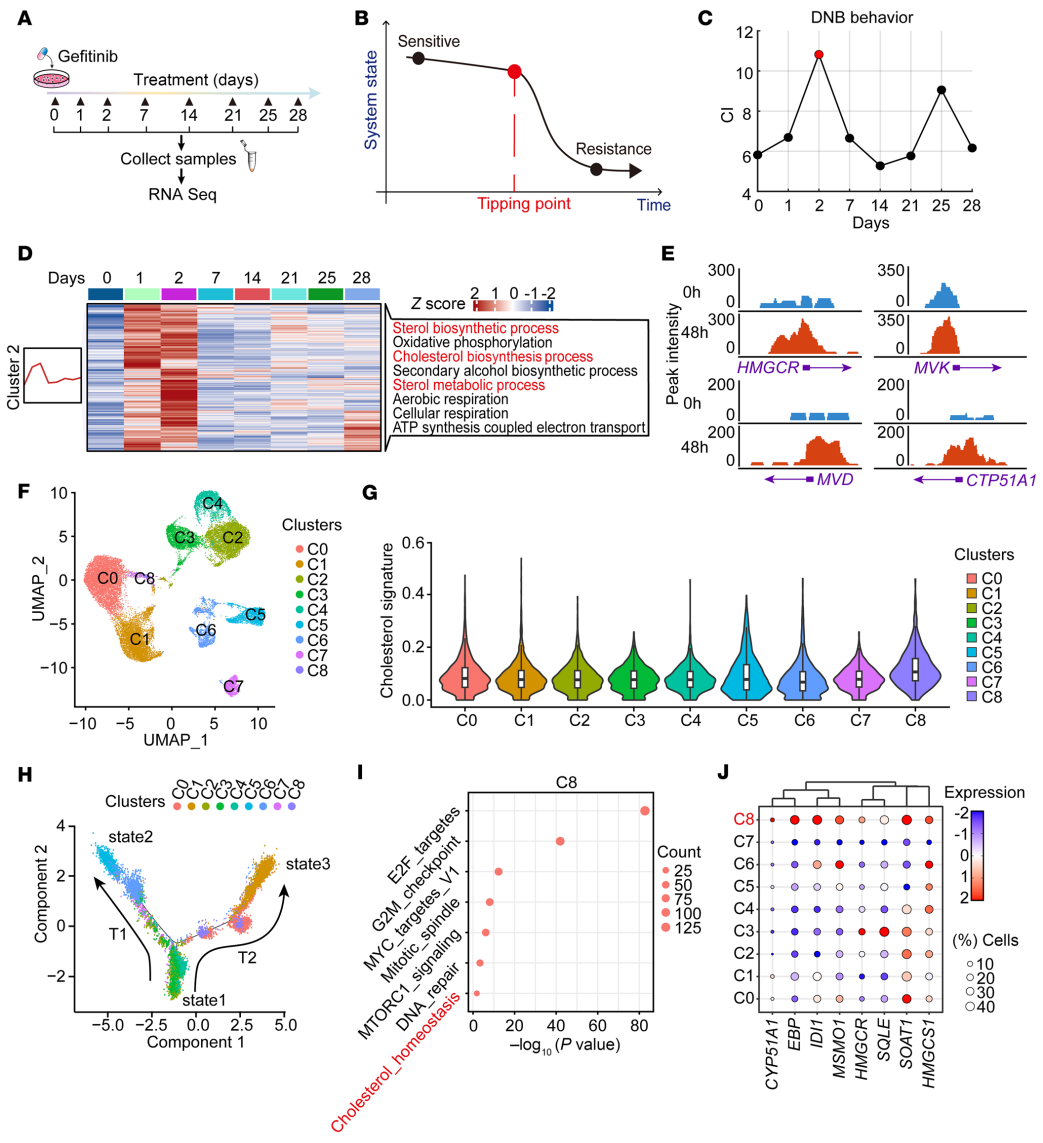
Targeted therapy activates cholesterol biosynthesis. (**A**) Schematic illustration of the experimental design for RNA-Seq of PC9 cells treated with gefitinib for the indicated times. (**B**) A schematic diagram of the system-state transition during the development of acquired drug resistance. (**C**) A CI for quantifying the TP of the system state. The peak of CI at day 2 indicates the TP. (**D**) Heatmap showing averaged signature enrichment scores of C2. (Left) The line chart represents the patterns of dynamic changes in DEGs, using Mfuzz. (Right) The biological processes in the black-outlined box are significantly enriched pathways in C2. Red text indicates the pathways of interest. (**E**) Chromatin accessibility around HMGCR, MVA kinase (MVK), MVA diphosphate decarboxylase (MVD), and the CTP51A1 promoter region in PC9 cells treated with gefitinib for 0 and 48 hours. (**F**) Uniform Manifold Approximation and Projection (UMAP) visualization of 5 paired human lung cancer specimens before and after afatinib therapy labeled with Seurat clusters. (**G**) Cholesterol signature scores of the 9 cell clusters from the 5 paired human lung cancer specimens. (**H**) Pseudotime ordering of the 9 cell clusters with Monocle 2. (**I**) Dot plot showing the Gene Ontology enrichment with HALLMARK pathway in C8. (**J**) Dot plot showing expression of cholesterol biosynthesis–related genes across different cell clusters. Dot diameter indicates the proportion of cells expressing a given gene; color indicates the relative expression level.

To uncover the temporal transcriptomic dynamics of lung cancer cells receiving targeted therapy, we treated PC9 cells with TKI in vitro and analyzed the RNA-Seq data of these cells at different drug-responsive states: sensitive (0–2 days), dormant (2–7 days), and resistant (7–28 days) (Figure 1A and Supplemental Figure 1D). We hypothesized that the development of TKI resistance is a nonlinear dynamic process: a drastic switch occurs once the system state approaches the critical threshold, termed the tipping point (TP) (15). Using dynamic network biomarker (DNB) analysis (16, 17), we identified the TP via genome-wide expression analysis of PC9 samples at different time points (Figure 1, B and C). Mfuzz analysis (18) revealed 8 distinct clusters based on the changes of gene expression profiles accompanying drug-resistance acquisition (Supplemental Figure 1E). Of note, cholesterol biosynthesis process was significantly enriched in cluster 2 (C2) at the TP (Figure 1D). This was further confirmed by the assay for transposase-accessible chromatin using sequencing (ATAC-Seq) data showing increased chromatin accessibility of multiple cholesterol biosynthesis–related gene promoters (namely, *HMGCR*, *MVK*, *MVD*, and *CTP51A1*) approaching the TP (Figure 1E and Supplemental Figure 1G). These findings indicate cholesterol synthesis might contribute to cancer dormancy and drug resistance.

We next evaluated the clinical relevance of cholesterol biosynthesis in EGFR-TKI–treated lung cancer. To this end, we performed single-cell RNA-Seq (scRNA-Seq) of 5 paired human EGFR-mutant lung cancer specimens before and after afatinib therapy. Using the InferCNV algorithm (19), we analyzed a total of 25,560 cancer cells and classified them into 9 subpopulations through unsupervised clustering (20) (Figure 1F and Supplemental Figure 1, H–K). Cluster 8 (C8) stood out as having the most significant enrichment of the cholesterol biosynthesis signature (Figure 1G). Trajectory analysis using Monocle (21) further classified all the cancer cells into 3 different states (Figure 1H and Supplemental Figure 1H). Based on this, 2 major trajectories were predicted through unsupervised pseudotime ordering: trajectory 1 (T1) and trajectory 2 (T2) (Figure 1H). C8 was mainly located at the transition route of T2 (Figure 1H). Further analyses showed that multiple cholesterol biosynthesis–related genes were highly expressed in C8 (Figure 1, I and J). PAGE analysis of the T2 trajectory revealed a dynamic and coordinated transcriptional program along this evolution route (Supplemental Figure 1, L and M). Several pathways progressively enriched along T2, such as epithelial-mesenchymal transition signaling, have been linked to TKI resistance (22). In addition, transcription factor (TF) analysis revealed progressive upregulation of *JUN* and *NFκBA* along T2 (Supplemental Figure 1N), both of which have been implicated in drug resistance (23, 24). These findings indicate a coordinated adaptive route by which cancer cells, under TKI pressure, progressively activate resistant programs, with C8 exhibiting enhanced cholesterol biosynthesis and serving as a potential transitional state in this process. These observations consistently point to the potential role of cholesterol in the link to EGFR-TKI resistance acquisition.

### Cholesterol is important for cancer cell survival.

**Figure 2:**
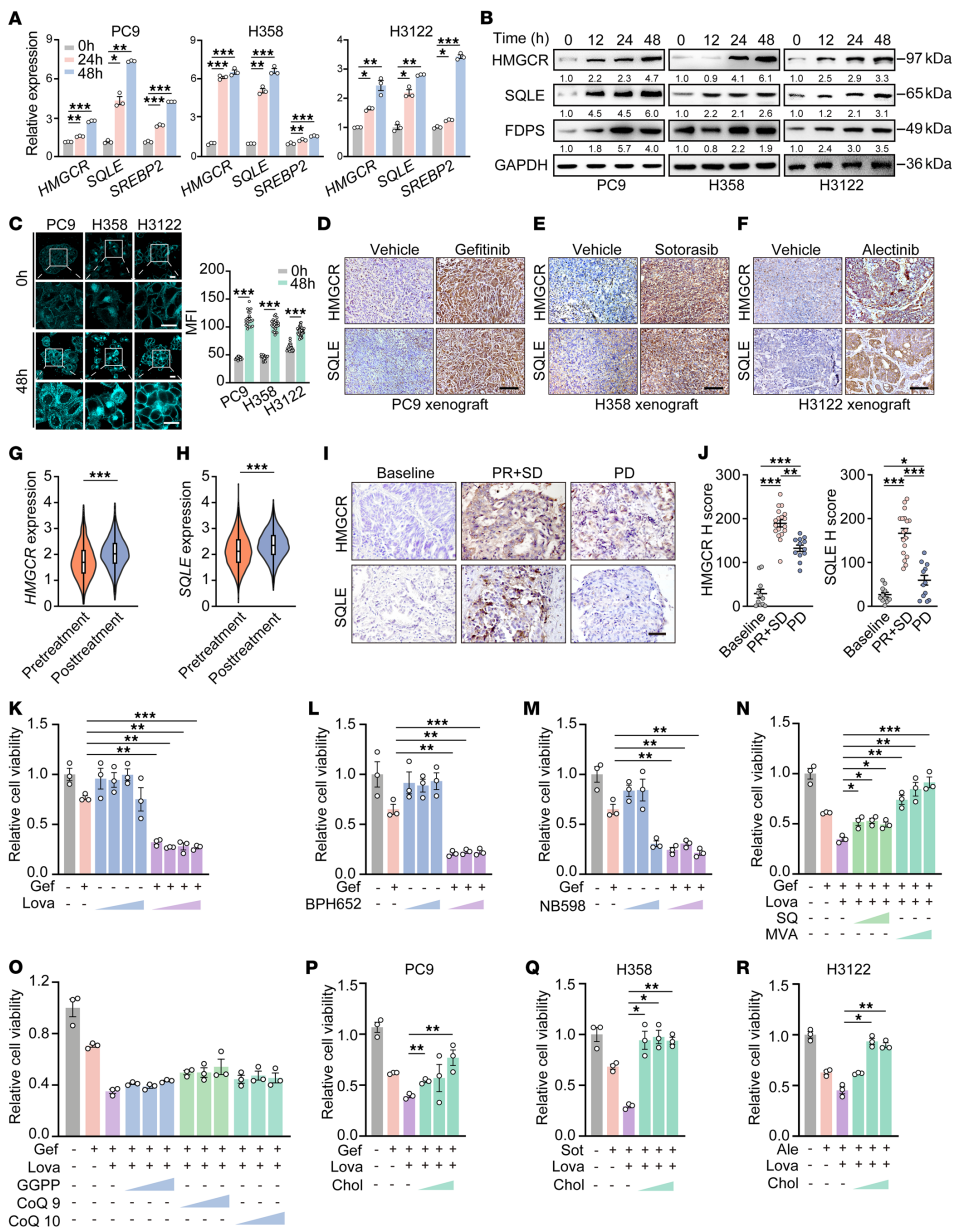
Cholesterol promotes lung cancer cell survival during targeted therapy. (**A**) Relative mRNA levels of *SREBP2*, *HMGCR*, and *SQLE* in PC9, H358, and H3122 cells treated with targeted therapy (gefitinib, sotorasib, and alectinib, respectively). (**B**) Western blot analysis of HMGCR, SQLE, and FDPS in these cell lines after targeted therapies. (**C**) Filipin staining of these cell lines after targeted therapies. Scale bar: 10 μm. (Right) Quantification of MFI of filipin. Each dot represents per cell. (**D**–**F**) Representative HMGCR and SQLE IHC staining in PC9, H358, and H3122 xenografts. Scale bar: 50 μm. (**G** and **H**) Violin plots depicting *HMGCR* or *SQLE* expression in scRNA-Seq data from 5 paired clinical samples. (**I** and **J**) Representative HMGCR and SQLE IHC staining and H-score quantification in EGFR-mutant clinical samples. Scale bar: 50 μm. Baseline (*n* = 11); PR plus stable disease (PR+SD) (*n* = 18); PD (*n* = 12). (**K**–**M**) Viability of PC9 cells treated with gefitinib (Gef) combined with lovastatin (Lova) (0.5–5 μM), BPH652 (50–200 μM) (**L**), or NB598 (25–100 μM) (**M**) for 48 hours. (**N**) Viability of PC9 cells treated with gefitinib, combined with lovastatin (5 μM), or with lovastatin plus squalene (SQ) (0.125, 0.25, or 0.5 μM) or MVA (0.25–1 mM) for 48 hours. (**O**) Viability of PC9 cells treated with gefitinib, combined with lovastatin, or with lovastatin plus geranylgeranyl pyrophosphate (GGPP) (0.5–2 μM), coenzyme Q9 (CoQ9) (2.5–10 μM), or coenzyme Q10 (CoQ10) (2.5–10 μM) for 48 hours. (**P**–**R**) Viability of these cell lines treated with targeted therapies, combined with lovastatin, or with lovastatin plus MβCD-coated cholesterol (Chol) (2.5–10 μg/mL) for 48 hours. Data in **A**–**C** and **K**–**R** represent 1 representative experiment of 3 independent replicates. For Western blot analysis, GAPDH served as the internal control. *P < 0.05, **P < 0.01, ***P < 0.001 by 1-way ANOVA with Dunnett’s multiple comparisons test (**A** and **J**–**R**); 2-tailed unpaired Student’s *t* test (**C** and **G**–**H**). Data are reported as mean ± SEM. Ale, alectinib; Sot, sotorasib.

We further found that enzymes involved in cholesterol biosynthesis, such as HMG-CoA reductase (*HMGCR*), squalene epoxidase (*SQLE*), farnesyl diphosphate synthase (*FDPS*), acetyl-CoA acetyltransferase 2 (*ACAT*), HMG-CoA synthase (*HMGCS*), MVA kinase (*MVK*), MVA diphosphate decarboxylase (*MVD*), isopentenyl-diphosphate delta isomerase 1 (*IDI1*), and cytochrome P450 family 51 subfamily A member 1 1 (*CYP51A*), were significantly upregulated upon TKI treatment, whereas the levels of enzymes from the branch pathway, such as geranylgeranyl diphosphate synthase 1 and farnesyltransferase were downregulated (Figure 2A and Supplemental Figure 2, A–C). Importantly, we found that SREBP2, a key regulator of cholesterol biosynthesis, along with its cleaved form N-terminal SREBP2 (25), were also upregulated and translocated to the cell nucleus, indicative of SREBP2 activation (Figure 2A and Supplemental Figure 2, D–F). HMGCR, SQLE, and FDPS protein levels increased in a time-dependent manner with TKI treatment (Figure 2B). These findings were also observed in other cell lines with KRAS mutation and ALK fusion (e.g., H358 cells [KRAS^G12C^]; H3122 cells [EML4-ALK fusion]). Sotorasib treatment in H358 cells and alectinib treatment in H3122 cells upregulated HMGCR and SQLE at both mRNA and protein levels, along with increased FDPS protein levels (Figure 2, A and B). These data demonstrate that the activation of cholesterol biosynthesis is a common response of cancer cells receiving targeted therapy.

Filipin staining is commonly used to detect free cholesterol in cells and tissues (26). Here, filipin staining further revealed the increase of cholesterol contents in cancer cells with targeted therapy (Figure 2C).

We next performed in vivo experiments and established H358 and H3122 xenograft tumors for sotorasib and alectinib treatments, respectively. These xenograft tumors initially responded to molecular targeted therapy quite well, but the tumors regrew quickly (Supplemental Figure 2, G and H). IHC staining showed increased protein levels of HMGCR and SQLE in drug-treated tumors compared with a control (Figure 2, D–F, and Supplemental Figure 2, I–K). These findings were also confirmed in the clinical setting. Our scRNA-Seq data revealed a higher expression of *HMGCR* and *SQLE* in the posttreatment group compared with the pretreatment group (Figure 2, G and H).

We further analyzed an independent cohort of 41 patient samples, containing 11 baseline samples, 18 partial response (PR) or stable disease samples, and 12 progressive disease (PD) samples. Consistently, protein levels of HMGCR and SQLE were significantly higher in post-TKI treatment samples (Figure 2, I and J).

We next treated PC9 cells with TKI in combination with various cholesterol synthesis inhibitors, including the HMGCR inhibitor lovastatin, and the SQLE inhibitors NB598 or BPH652 (Figure 2, K–M). Notably, combining gefitinib with any of these cholesterol synthesis inhibitors significantly suppressed PC9 cell survival (Figure 2, K–M). We also performed CRISPR/Cas9-mediated knockout of HMGCR in PC9, H358, and H3122 cells. Consistent with the effects of lovastatin, HMGCR knockout markedly sensitized these cells to their respective targeted therapies, as evidenced by significantly reduced cell viability compared with scramble controls (Supplemental Figure 2, L–N). These results provide direct genetic evidence that HMGCR activity is essential for cancer cell survival upon TKI treatment. Individual supplementation with those metabolites directly involved in cholesterol biosynthesis such as MVA, squalene, and cholesterol reversed the inhibitory effects of lovastatin, whereas those metabolites from the branch pathways including geranylgeranyl pyrophosphate, coenzyme Q9, or coenzyme Q10 could not (Figure 2, N–P). Similarly, lovastatin treatment enhanced the inhibitory effects of sotorasib in H358 cells and alectinib in H3122 cells, which could also be reversed by cholesterol supplementation (Figure 2, Q and R). Moreover, cholesterol supplementation significantly reduced combination treatment–induced apoptosis (Supplemental Figure 2, O–R). These results suggest that the increase of cholesterol biosynthesis promotes the survival of lung cancer cells in response to targeted therapies.

### PERK-eIF2α signaling contributes to cholesterol biosynthesis.

**Figure 3:**
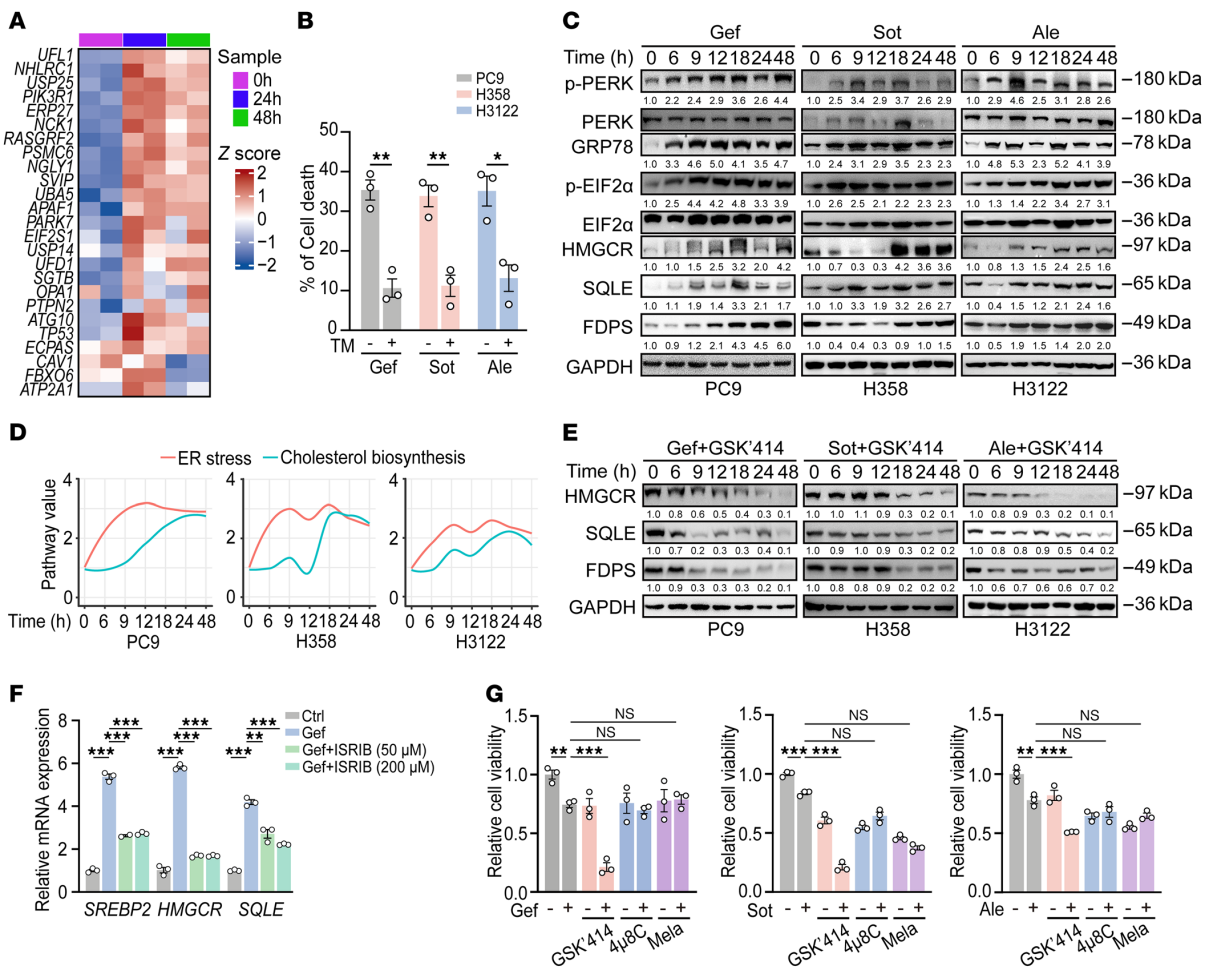
PERK/eIF2α signaling contributes to cholesterol biosynthesis triggered by targeted therapy. (**A**) Heatmap based on RNA-Seq data used in Figure 1A showing expression of ER stress signature genes in PC9 cells after gefitinib (Gef) treatment (*n* = 2 biological replicates/group). (**B**) Cell death analysis in PC9, H358, and H3122 cells treated with targeted therapies alone or pretreated with tunicamycin (TM) (1 μg/mL) for 48 hours. (**C**) Western blot analyses of indicated proteins in PC9, H358, and H3122 cells treated with targeted therapies for the indicated times. (**D**) Fitted pathway activity curves based on quantified expression levels of proteins from (**C**). The ER stress pathway (red) is represented by p-PERK, p-EIF2α, and GRP78, and the cholesterol biosynthesis pathway (blue) is represented by HMGCR, SQLE, and FDPS. Protein expression values were quantified, and curves were generated using the LOESS method (100) to show temporal trends in pathway activation in PC9, H358, and H3122 cells after treatment with targeted therapies at the indicated times. (**E**) Western blot analyses of HMGCR, SQLE, and FDPS in cells cotreated with targeted therapies and GSK2606414 (GSK’414) (10 μM) for the indicated times. (**F**) Real-time PCR detection of *SREBP2*, *HMGCR*, and *SQLE* mRNA levels in PC9 cells treated with gefitinib alone or in combination with integrated stress response inhibitor (ISRIB) (50 or 200 μM) for 48 hours. (**G**) Relative viability of PC9, H358, and H3122 cells treated with targeted therapies alone, or in combination with GSK’414 (5 μM), 4μ8C (15 μM), or melatonin (Mela) (2 mM) for 48 hours. Data in **B**, **E**, **F**, and **G** represent 1 representative result of 3 independent experiments. For Western blot analysis, GAPDH served as the internal control. *P < 0.05, **P < 0.01, ***P < 0.001 by 1-way ANOVA with Dunnett’s multiple comparisons test (**F** and **G**); 2-tailed unpaired Student’s *t* test (**B**). Data are represented as mean ± SEM. Ale, alectinib; Sot, sotorasib.

Next, we sought to elucidate the mechanisms underlying cholesterol biosynthesis in response to targeted therapies. RNA-Seq data analyses revealed a marked upregulation of unfolded protein response–related (UPR-related) genes (Figure 3A), indicative of potential UPR activation and ER stress induction. ER stress is known to promote cell survival during EGFR-TKI treatment (27), induce SREBP1/2 activation, and enhance cellular cholesterol accumulation (28–32). In line with earlier reports (33), we found that treatment with the UPR agonist tunicamycin significantly reduced targeted therapy–induced cell death (Figure 3B). Consistently, we observed an upregulation of glucose-regulated protein 78 (GRP78) (34) in all 3 cell lines upon targeted therapy (Figure 3C).

We then examined the 3 known major UPR sensors: activating transcription factor 6 (ATF6), inositol-requiring enzyme 1 (IRE1), and protein kinase RNA–like endoplasmic reticulum kinase (PERK) (35). We found that total level of ATF6 was downregulated and its cleaved form was undetectable (Supplemental Figure 3A). Additionally, spliced *XBP1* (*XBP1-s*), a marker of the IRE1 pathway (36), was also undetectable after drug treatment (Supplemental Figure 3B). In contrast, the levels of phosphorylated PERK (p-PERK) and phosphorylated eIF2α (p-eIF2α) were notably upregulated following molecular targeted therapy, indicative of preferential activation of the PERK-eIF2α axis (Figure 3C).

We then temporally analyzed the expression patterns of UPR and cholesterol synthesis–related genes, using p-PERK, p-eIF2α, and GRP78 as ER stress reporters, and HMGCR, SQLE, and FDPS as cholesterol biosynthesis reporters. We found that ER stress was activated earlier than cholesterol biosynthesis during drug treatment (Figure 3D), indicating that ER stress may promote cholesterol biosynthesis upon targeted therapy.

To determine the role of the PERK-eIF2α axis in cholesterol biosynthesis, we treated cancer cells with GSK2606414 (a PERK inhibitor) and observed a notable abrogation of upregulation of HMGCR, SQLE, and FDPS induced by targeted therapies (Figure 3E). Consistently, treatment with integrated stress response inhibitor (a p-eIF2α inhibitor) also reversed targeted therapy–induced upregulation of *SREBP2*, *HMGCR*, and *SQLE* (Figure 3F). These results suggest the PERK/eIF2α axis contributes to cholesterol biosynthesis downstream of ER stress in response to targeted therapy. Moreover, GSK2606414 treatment significantly sensitized cells to targeted therapy (Figure 3G).

A recent study highlighted the functional importance of the reactivation of the IRE1α branch of the UPR in promoting KRAS-inhibitor resistance (37). We found that 4μ8C treatment (an IRE1 inhibitor) could not reverse targeted therapy–induced upregulation of *HMGCR* and *SQLE* and failed to sensitize cells to targeted therapy (Figure 3G and Supplemental Figure 3C). Melatonin (an ATF6 inhibitor) treatment also failed to sensitize cells to targeted therapy (Figure 3G). These data collectively support the notion that targeted therapy–induced ER stress contributes to cholesterol biosynthesis and promotes cell survival through activating the PERK/eIF2α axis. As a result, targeting this axis suppresses cholesterol biosynthesis and sensitizes lung cancer cells to targeted therapy.

### Cholesterol biosynthesis promotes AKT activation.

**Figure 4:**
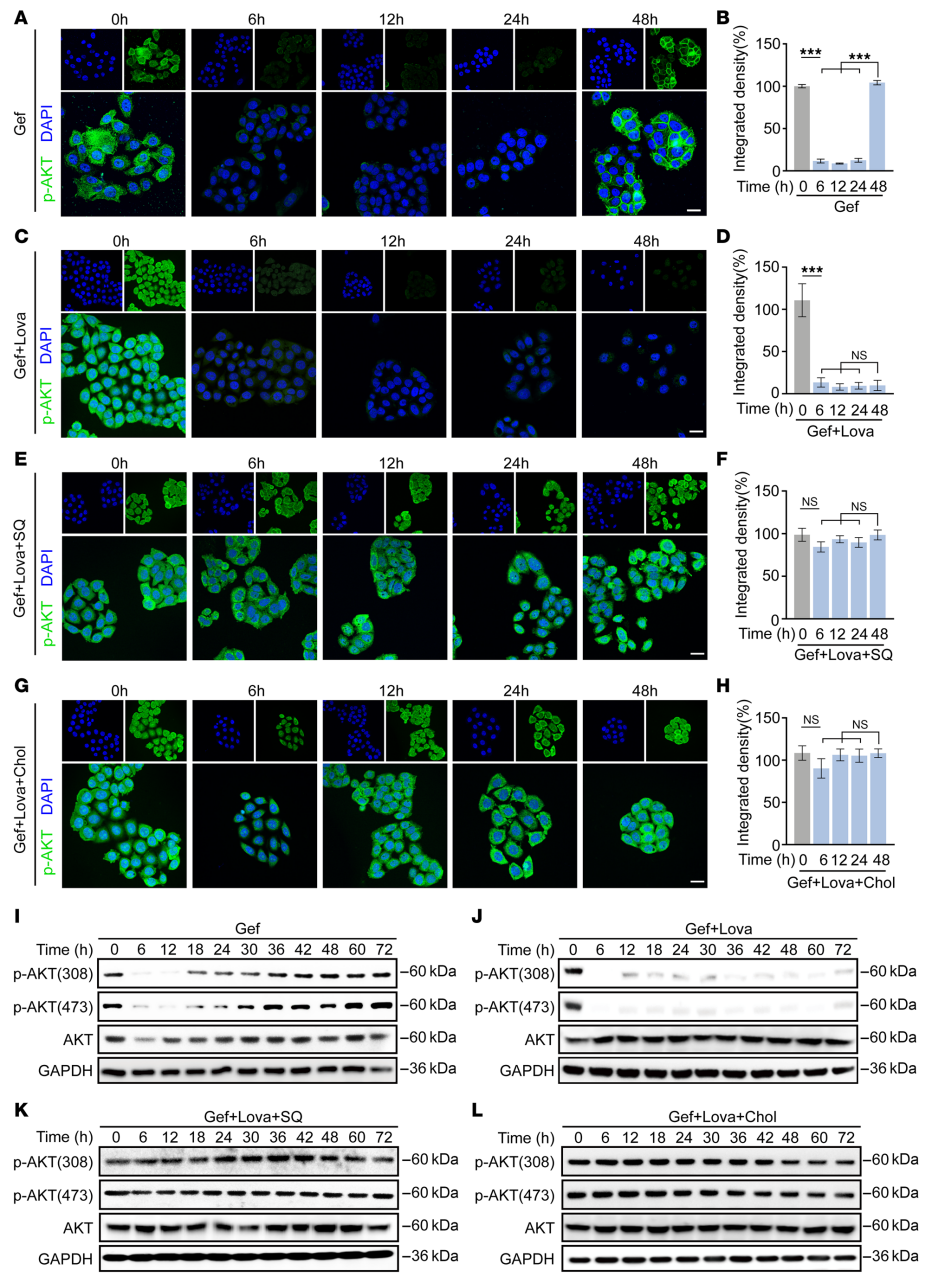
Cholesterol biosynthesis promotes AKT activation. (**A** and **B**) Representative IF staining (**A**) and quantification (**B**) of p-AKT Ser473 in PC9 cells treated with gefitinib (Gef) for the indicated times. (**C** and **D**) Representative IF staining (**C**) and quantification (**D**) of p-AKT Ser473 in PC9 cells treated with gefitinib in combination with lovastatin (Lova) (5 μM) for the indicated times. (**E** and **F**) Representative IF staining (**E**) and quantification (**F**) of p-AKT Ser473 in PC9 cells treated with gefitinib in combination with lovastatin (5 μM) and squalene (SQ) (0.5 μM) for the indicated times. (**G** and **H**) Representative IF staining (**G**) and quantification (**H**) of p-AKT Ser473 in PC9 cells treated with gefitinib in combination with lovastatin (5 μM) and MβCD-coated cholesterol (10 μg/mL) for the indicated times. (**A**, **C**, **E**, and **G**) p-AKT is shown in green, and nuclei are stained with DAPI (blue). Scale bar: 10 μm. (**I**–**L**) Western blot analysis of p-AKT Ser308 and Ser473 in PC9 cells treated with gefitinib (**I**), or in combination with lovastatin (5 μM) (**J**), or in combination with lovastatin (5 μM) and squalene (0.5 μM) (**K**) or MβCD-coated cholesterol (Chol) (10 μg/mL) (**L**) for the indicated times. Data represent 1 representative result of 3 independent experiments. GAPDH was used as an internal control. ***P < 0.001 by 1-way ANOVA with Dunnett’s multiple comparisons test (**B**, **D**, **F**, and **H**). Data are represented as mean ± SEM.

Cholesterol is a key component of membrane lipid rafts, and its accumulation in lipid rafts can regulate membrane-bound proteins and activate signaling cascades, particularly activating AKT signaling, which promotes cell survival and confers resistance to apoptosis (38). We then examined the AKT signaling in PC9 cells after TKI treatment. Immunofluorescence (IF) staining showed that the level of phosphorylated-AKT (p-AKT) was reduced at 24 hours after TKI treatment but significantly rebounded at 48 hours (Figure 4, A and B). We further found that TKI treatment alone elicited rapid (<6 hours) and sustained (>72 hours) inhibition of EGFR and S6K phosphorylation (Supplemental Figure 4, A and B). However, TKI monotherapy only transiently suppressed AKT activation (<24 hours), with its reactivation occurring at 48 hours after drug treatment (Figure 4, A and B), indicative of an EGFR-independent reactivation of AKT. Consistently, combination therapy with TKI and lovastatin effectively blocked AKT reactivation and achieved sustained inhibition of this pathway (Figure 4, C and D). Notably, supplementation with squalene or cholesterol almost completely reversed the inhibitory effect of lovastatin on AKT reactivation (Figure 4, E–H).

We further assessed p-AKT expression levels by Western blot analysis and observed that targeted monotherapy similarly induced feedback activation of p-AKT (Figure 4I and Supplemental Figure 4C), whereas this feedback activation was markedly suppressed when targeted therapy was combined with lovastatin (Figure 4J and Supplemental Figure 4D). Moreover, supplementation with squalene and cholesterol profoundly restored p-AKT levels in lovastatin-treated cells without affecting p-EGFR levels (Figure 4, K and L, and Supplemental Figure 4, E–J). These loss-of-function and gain-of-function experiments consistently demonstrate the requirement of cholesterol biosynthesis in the feedback activation of AKT.

To validate the functional importance of AKT reactivation in this setting, we tested the AKT-specific inhibitor AKT inhibitor VIII (39) in PC9, H358, and H3122 cells. The combination of targeted therapy with AKT inhibitor VIII had comparable efficacy to the TKI plus lovastatin combination in reducing cell viability (Supplemental Figure 4, K–M). These findings indicate that targeted therapy–induced cholesterol biosynthesis can activate AKT signaling, thereby promoting cancer cell survival to maintain cancer dormancy.

### Dormant cancer cells have high levels of cholesterol and AKT activation.

**Figure 5:**
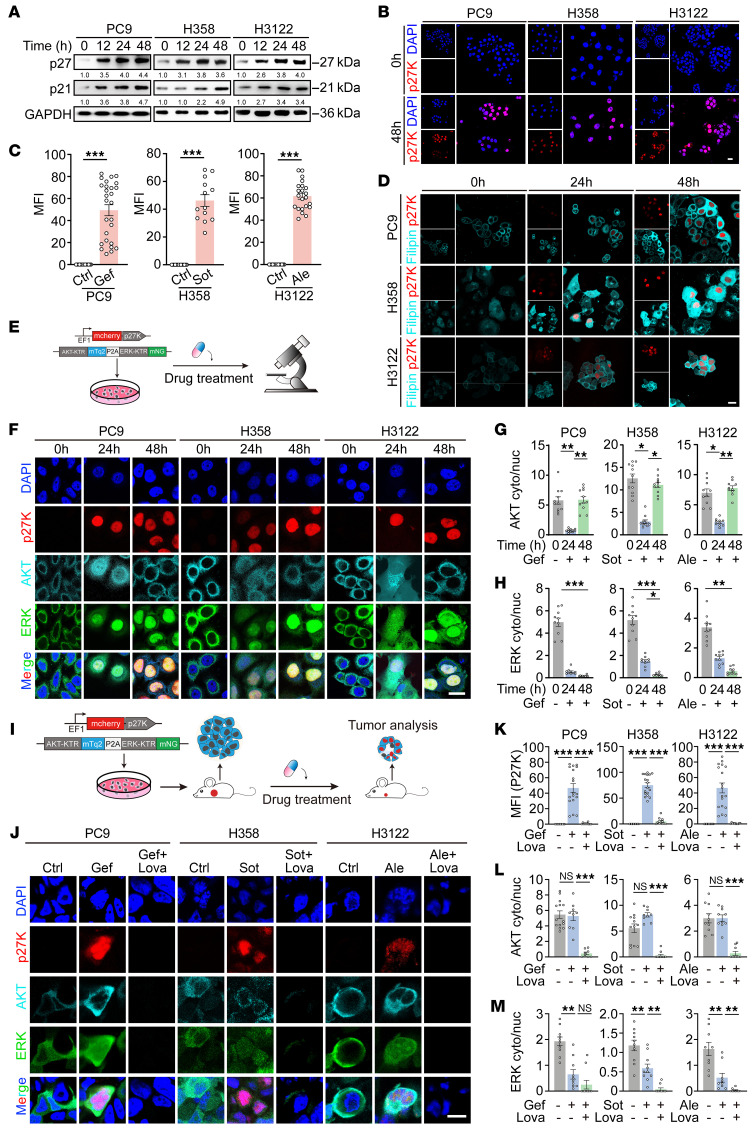
Dormant cancer cells have high levels of cholesterol and AKT activation. (**A**) Western blot analysis of p21 and p27 in PC9, H358, and H3122 cells treated with targeted therapies. (**B**) Representative IF images of mcherry-p27K (red) and DAPI (blue) in these cell lines after exposure to targeted therapies. Scale bar: 10 μm. (**C**) Quantification of MFI of mcherry-p27K from (**B**). (**D**) Filipin staining in cells expressing mcherry-p27K after targeted therapies. Scale bars: 10 μm. (**E**) Schematic illustration of the in vitro experimental design. (**F**) Representative IF images of cells co-expressing mcherry-p27K (red) and AKT-KTR (cyan) or ERK-KTR (green) for the indicated times. Scale bars: 10 μm. (**G** and **H**) AKT (**G**) or ERK (**H**) pathway activity, measured as the AKT/ERK-KTR cytoplasmic-to-nuclear intensity ratio (cyto/nuc) from (**F**). A higher ratio indicates greater pathway activity. (**I**) Schematic illustration of the xenograft study design. Mice bearing tumors from cells co-expressing mcherry-p27K and AKT/ERK-KTR were treated daily with targeted therapy (25 mg/kg gefitinib [Gef], 30 mg/kg sotorasib {Sot], or 30 mg/kg alectinib [Ale]) alone or in combination with 10 mg/kg lovastatin [Lova]. (**J**) Representative IF images of xenograft tissue showing staining with mCherry-p27K (red), AKT-KTR (cyan), ERK-KTR (green), and DAPI (blue). Scale bar: 10 μm. (**K**) Quantification of mCherry-p27K MFI from (**J**). (**L** and **M**) AKT (**L**) or ERK (**M**) pathway activity in xenografts, measured as the AKT/ERK-KTR cytoplasmic-to-nuclear ratio from (**J**). Data in **A**, **B**, **D**, **F**, and **J** represent 1 representative result of 3 independent experiments. Fluorescence intensity was analyzed using Image J. *P < 0.05, **P < 0.01, ***P < 0.001 by 1-way ANOVA with Dunnett’s multiple comparisons test (**G**, **H**, and **K**–**M**); 2-tailed unpaired Student’s *t* test (**C**). Data are represented as mean ± SEM. Ctrl, control.

Previous studies have shown that cancer cell persisters often enter dormancy upon drug exposure, which enables them to survive therapy (40–42). Consistently, we observed a significant upregulation of p27 and p21, 2 well-known biomarkers of dormancy (43), after targeted therapy (Figure 5A). Using mcherry-p27K as indicator of dormancy (44), we observed a notable dormancy after drug treatment (Figure 5, B and C). Further analysis revealed that the levels of p27K and filipin staining were positively correlated (Figure 5D and Supplemental Figure 5, A–C), indicating dormant cancer cells induced by targeted therapy have elevated cholesterol levels.

To further visualize the activities of AKT and ERK signaling in lung cancer cells after being exposed to targeted therapies, we used a kinase translocation reporter (KTR) (45), which can provide real-time monitoring of kinase activities via calculation of the fluorescence nuclear-to-cytoplasmic ratio (46, 47) (Figure 5E). Our results confirmed the reactivation of AKT, but not ERK, in P27K-positive cells after drug treatment (Figure 5, F–H). The inactivation of ERK in dormant cells was in agreement with the notion that ERK activation is typically associated with cell proliferation (48, 49). Moreover, we transplanted the KTR cells with p27K-mcherry reporter into nude mice to generate xenograft models for targeted therapy (Figure 5I and Supplemental Figure 5D). In line with the in vitro data, targeted therapy induced a marked increase in P27K-positive cells with high AKT activity and low ERK activity (Figure 5, J–M). Lovastatin treatment effectively blocked AKT activation and eliminated P27K-positive cells (Figure 5, J–M). These findings demonstrate that targeting cholesterol biosynthesis could be a potential strategy for eliminating dormant cells induced by targeted therapy.

### Combination of targeted therapy with lovastatin and alisertib has synergistic antitumor activity.

**Figure 6:**
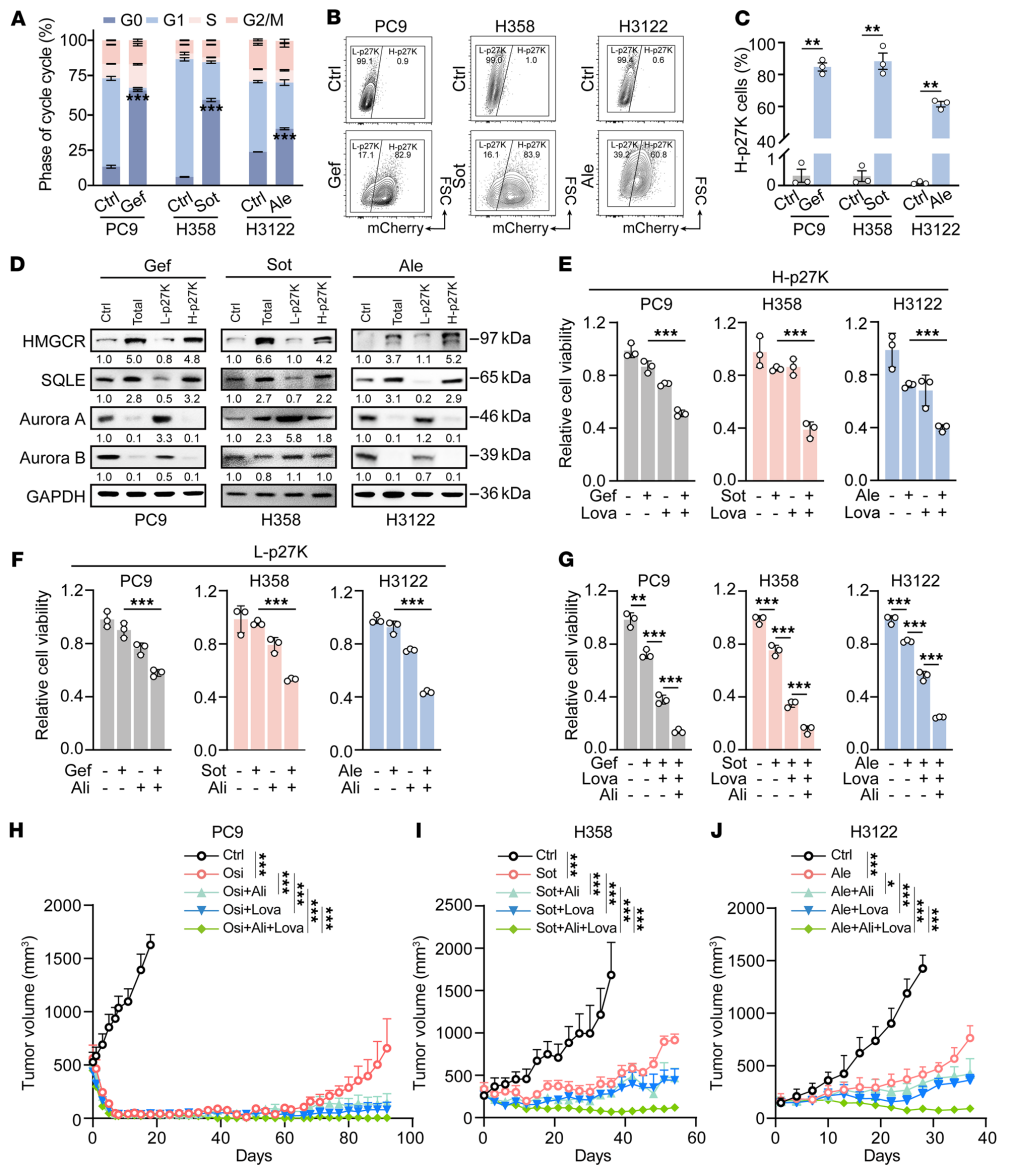
Combination of targeted therapy with lovastatin and alisertib shows synergistic antitumor activity. (**A**) Proportion of cells in each cell cycle phase after treatment with targeted therapies for 48 hours. (**B**) Representative flow cytometry analysis of p27K levels in DAPI-negative PC9, H358, and H3122 cells expressing mcherry-p27K fusion protein treated with targeted therapies for 48 hours. The plots are gated into 2 regions: low-p27K (L-p27K) and high-p27K (H-p27K) subpopulations. The subpopulations were then subjected to Western blot analysis and cell viability detection. (**C**) Quantification of the H-p27K subpopulation of PC9, H358 and H3122 cells in panel (**B**). (**D**) Western blot analysis of HMGCR, SQLE, aurora A, and aurora B in PC9, H358, and H3122 cells treated with targeted therapy. (**E** and **F**) Relative viability of H-p27K (**E**) or L-p27K (**F**) subpopulations in PC9, H358, and H3122 cells treated with targeted therapies, or in combination with lovastatin (Lova) (5 μM) or Alisertib (Ali) (5 nM) for 48 hours. (**G**) Relative viability of PC9, H358, and H3122 cells treated with targeted therapies, or in combination with lovastatin, alisertib, or both. (**H**–**J**) Tumor growth of PC9 (**H**), H358 (**I**), or H3122 (**J**) xenograft treated with vehicle, targeted therapies, or in combination with lovastatin, alisertib, or both. *n* = 8 mice per group. Drugs were administrated by daily oral gavage in the following doses: osimertinib (Osi), 5 mg/kg; sotorasib (Sot), 30 mg/kg; lovastatin (Lova), 10 mg/kg; alisertib (Ali), 10 mg/kg. Data in **A**, **B**, and **D**–**G** represent 1 representative result of 3 independent experiments. For Western blot analysis, GAPDH served as the internal control. *P < 0.05, **P < 0.01, ***P < 0.001 by 1-way ANOVA with Dunnett’s multiple comparisons test (**E**–**J**); 2-tailed unpaired Student’s *t* test (**A** and **C**). Data are represented as mean ± SEM. Ale, alectinib; Ctrl, control.

A previous study showed that although most cancer persisters initially remain dormant during drug treatment, a small subpopulation can reenter the cell cycle (50). Flow cytometry analysis revealed that a small subpopulation remained in the S and G2/M phase although most other cells were in the G0 phase (Figure 6A). This is consistent with previous studies showing high heterogeneity of cancer cells in responses to targeted therapy (51, 52). Flow cytometry analysis further revealed a bimodal distribution after drug treatment, with a major dormant cell subpopulation showing high p27K expression (~80% for PC9 and H358, and 60% for H3122 of the total population), and a small cycling subpopulation showing low p27K expression (Figure 6, B and C). These dormant cells expressed higher levels of HMGCR and SQLE and had elevated cholesterol content compared with the cycling cells (Figure 6D and Supplemental Figure 6A). Moreover, the dormant cells were sensitive to combined TKI and lovastatin treatment (Figure 6E).

Aurora kinase signaling has been implicated in drug-resistance in EGFR-mutant and KRAS-mutant cancers (12, 53, 54). We found that the cycling cells had increased aurora kinase A levels compared with dormant cells (Figure 6D). Consistently, the scRNA-Seq data of the 5 paired human lung cancer specimens showed that cancer cells with a low dormancy score (55) (the cycling subpopulation) expressed significantly higher levels of both *AURKA* and *AURKB* (Supplemental Figure 6B). Alisertib, an aurora kinase inhibitor, has recently been approved by the FDA as an orphan drug for the treatment of small cell lung cancer. We found that the combination of targeted therapy and alisertib led to synergistic inhibition of cycling cell survival (Figure 6F). Notably, the combination of targeted therapy and lovastatin failed to induce an obvious synergistic inhibitory effect in low-p27K-expression cells, and the combination of targeted therapy and alisertib similarly failed to show synergy in high-p27K-expression cells (Supplemental Figure 6, C and D). These reciprocal experiments support the notion that the synergistic inhibitory effect of cotreatment is mainly dependent on the expression of the respective targeted enzyme by lovastatin or alisertib.

Moreover, triple-targeting cholesterol biosynthesis, aurora kinase, and individual oncogenic drivers achieved the greatest growth-inhibitory effect in vitro when compared with targeted therapy alone or in combination with lovastatin (Figure 6G). Consistently, the triple-targeting therapy almost completely blocked PC9 tumor regrowth over 90 days, whereas the tumors in the osimertinib monotherapy group regrew after 60 days (Figure 6H). Tumor eradication rates were 88% (*n* = 7 of 8) in the triple-targeting group, 66% (*n* = 6 of 9) in the lovastatin plus osimertinib group, 33% (*n* = 3 of 9) in the alisertib plus osimertinib group, and 25% (*n* = 2 of 8) in the osimertinib monotherapy group. Similarly, in both the H358 and H3122 xenograft models, the triple-targeting regimen—sotorasib-lovastatin-alisertib combination for H358 and alectinib-lovastatin-alisertib combination for H3122—achieved sustained tumor regression with the greatest antitumor efficacy, whereas the respective monotherapies led to tumor regrowth (Figure 6, I and J).

Histological analysis of the H358 xenograft tumors showed that, compared with sotorasib monotherapy, dual-targeting treatments further suppressed proliferation and induced apoptosis, as evidenced by reduced Ki67 staining (a proliferation marker) and elevated cleaved-caspase 3 levels (an apoptosis marker) (Supplemental Figure 6E). Tumors from the triple-targeting group were too small (~20 mm³) for reliable histological analysis and were thus absent from staining analysis (Supplemental Figure 6E). In line with our earlier observation, sotorasib treatment significantly upregulated HMGCR protein levels (Supplemental Figure 6E). We noticed that lovastatin did not abrogate sotorasib-induced HMGCR upregulation, which is likely because lovastatin inhibits the enzymatic activity of HMGCR rather than its expression, as previously reported (56).

Importantly, no increased systemic and liver toxicity were observed in the triple-targeting group, as assessed by body weight and liver function tests in tumor-bearing and wild-type mice (Supplemental Figure 6, F–L). Collectively, these results suggest that combinational inhibition of both cholesterol synthesis and aurora kinase is effective in delaying acquired resistance to targeted therapies in lung cancer.

## Discussion

**Figure 7:**
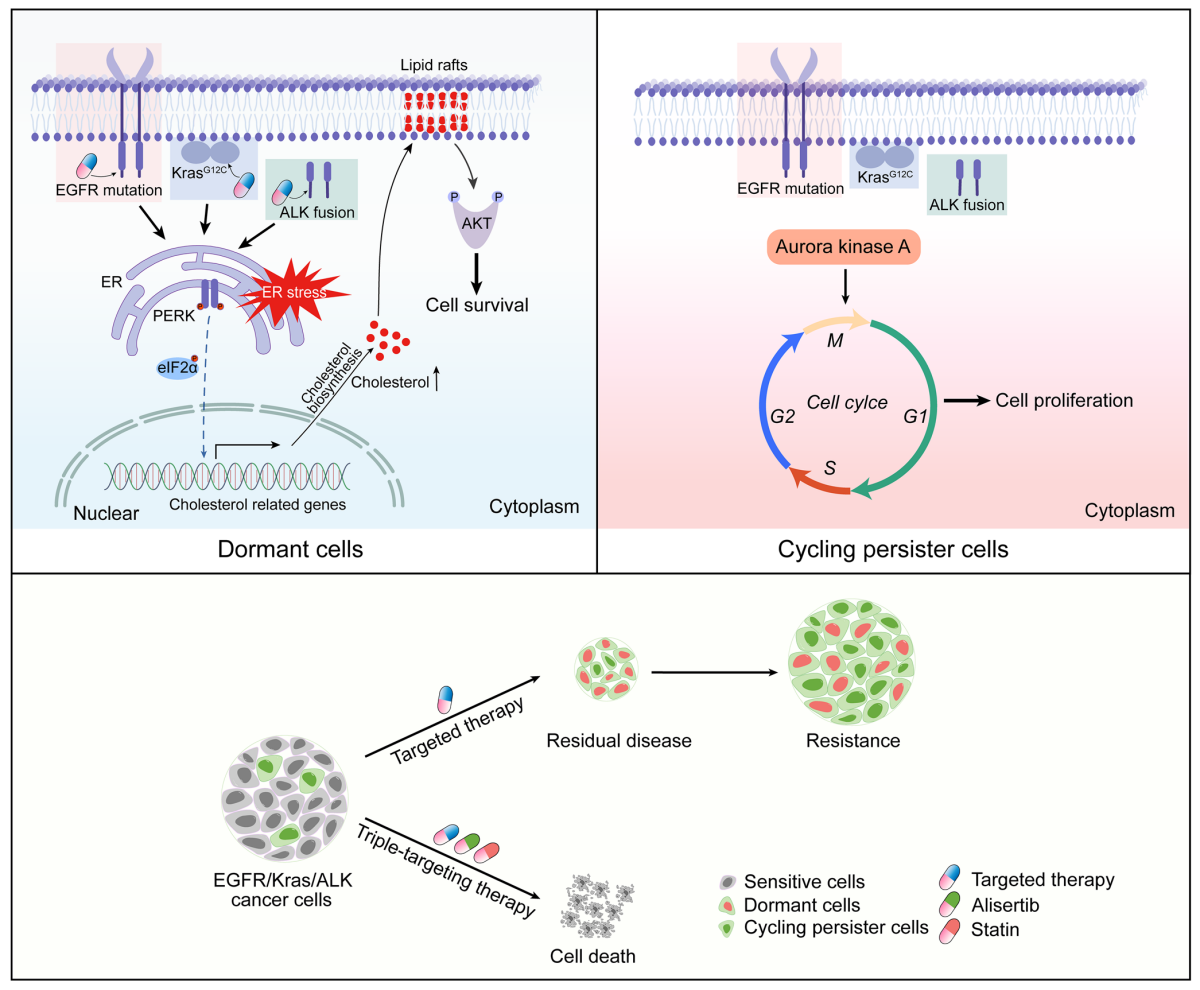
Working model. Molecular targeted therapy induces cancer dormancy through activating cholesterol biosynthesis via triggering the UPR, and increased cholesterol level facilitates AKT activation, thereby promoting cancer cell survival. In addition to dormant cancer cells, a small subset of cycling cells persists during molecular targeted therapy, which expresses high levels of aurora kinase A and remains highly sensitive to aurora kinase inhibitors. A triple-targeting strategy, which includes targeting cholesterol biosynthesis with statins to eliminate dormant cells, targeting aurora kinase with alisertib to inhibit cycling cells, and targeting individual oncogenic drivers, exerts a synergetic effect in the eradication of cancer cells.

Acquired resistance to molecular targeted therapy remains an important unsolved clinical challenge. A better understanding of the underlying mechanisms by which cancer cells evade initial killing in response to drug treatment or maintain dormancy after drug treatment is crucial for improving clinical outcome (57). Here, we identify a therapy-induced prosurvival program: the molecular targeted therapy first triggers the UPR activation and then activates cholesterol biosynthesis, which subsequently results in AKT activation and cancer cell survival, and eventually contributes to cancer dormancy. These findings might explain why there are few complete responses in clinic when patients receive molecular targeted therapy. Beside dormant cancer cells, we also identify a cycling cancer cell subpopulation that persists during molecular targeted therapy, expresses high levels of aurora kinase A, and has high sensitivity to aurora kinase inhibitors. Based on these data, we propose a triple-targeting strategy through combinational targeting of cholesterol biosynthesis to eliminate dormant cells, targeting aurora kinase to inhibit cycling cells, and targeting individual oncogenic drivers to improve the efficacy of molecular targeted therapy in the lung cancer clinic (Figure 7).

Increasing evidence has demonstrated that rapid, nongenetic, adaptive programs often precede and enable the emergence of genetic mutation–driven resistance (58). To capture the early adaptive programs that allow a subset of cancer cells to survive initial TKI treatment, we performed DNB analysis to dissect the temporal transcriptomic dynamics in lung cancer cells following targeted therapy. Our data identify cholesterol biosynthesis as a key process significantly enriched at the critical transition point, highlighting its role in early adaptive responses. Dysregulated cholesterol metabolism is commonly observed in multiple cancers, including lung cancer, and links to therapeutic resistance (59–61). We find that UPR and ER stress acts as a major upstream regulator in promoting cholesterol biosynthesis and cell survival after molecular targeted therapy via activating the PERK/eIF2α axis. This finding is in line with a previous study that identified UPR as a major adaptive mechanism in dormant cells, which enables them to withstand various stresses (62). UPR is known to attenuate translation through modulation of eIF2α and induce cell cycle arrest via the PERK pathway (63), thereby supporting cell survival under adverse conditions. Notably, the integrated stress response and its downstream signaling pathways have been demonstrated to contribute significantly to both KRAS-mutant lung cancer pathogenesis (64) and acquired resistance to KRAS inhibitors (37). For example, Lv et al. (37) showed that IRE1α is selectively reactivated in KRASi-resistant tumors and important for restoring proteostasis and promoting KRASi-resistant tumor growth. We demonstrate here that targeted therapy triggers the PERK/eIF2α axis to induce cholesterol biosynthesis, which, in turn, facilitates AKT activation and cancer cell survival. We did not observe a notable presence of *XBP1-s*, a marker of the IRE1 pathway, following targeted therapy. Moreover, treatment with 4μ8C, an IRE1 inhibitor, failed to sensitize cells to targeted therapy. We also found that 4μ8C treatment did not reverse targeted therapy–induced upregulation of HMGCR and SQLE. These results collectively demonstrate that the IRE1α/XBP1 axis is not involved in the targeted therapy–induced cholesterol biosynthesis and prosurvival program in our system. The discrepancies between the Lv et al. study (37) and our present study may stem from stage-specific events in response to targeted therapy. Lv et al. investigated KRAS inhibitor resistance using long-term treatments (>20 days) (37). In contrast, we aimed to dissect the mechanisms underlying the early stress responses associated with therapy-induced dormancy using a shorter treatment duration (~48 hours).

We further reveal that cholesterol-promoted AKT activation is an important downstream molecular event that facilitates dormant cancer cell survival under drug treatment. It is known that cholesterol enrichment in membrane lipid rafts promotes AKT recruitment and activation, thus facilitating cell survival and apoptosis resistance (65, 66). Our data show AKT activation plays a crucial role in promoting survival in dormant cells. It is well established that many pathways can reactivate AKT (68). While we cannot exclude the potential involvement of other alternative mechanisms contributing to AKT reactivation in this setting, our loss-of-function and gain-of-function experiments consistently support the notion that increased cholesterol content may be an important mechanism underlying TKI-induced AKT reactivation. Considering the complexity of cholesterol-mediated signaling cascades (68, 69), it is possible that multiple mechanisms might be involved in cholesterol-mediated dormant cell survival during targeted therapy, which requires future efforts to dissect the details.

We observed a positive correlation between the levels of p27K and filipin staining after targeted therapy, suggesting that dormant cancer cells in G0 phase have higher levels of cholesterol than cells in other phases in this setting. Although we did not systematically analyze changes in cholesterol levels in cells during different cell cycle stages, our findings support the functional importance of cholesterol in the survival and maintenance of dormant cancer cells following drug treatment. It will be interesting to determine whether the regulation of cholesterol levels is cell cycle or stimuli specific.

Statins are the most commonly used cholesterol-lowering agents in the clinic and exert antitumor properties in a variety of malignancies, including lung cancer (70–76). Despite these findings, a randomized phase II clinical trial showed that EGFR TKI plus statin combination therapy provides no significant survival benefit for previously treated patients with non–small cell lung cancer (NSCLC) (77). Because these patients’ disease has relapsed after chemotherapy and the majority are wild-type for EGFR, the potential efficacy of statins could be largely underestimated. Our results show that combined treatment with a statin as initial therapy for treatment-naive tumors could prevent the development of acquired resistance. These findings indicate the timing of statin administration and the selection of patient subgroups might be equally important when designing clinical trials. Efforts are needed to focus on well-designed clinical trials to validate the antitumor effects of statin adjuvant therapy in molecular targeted therapy.

After osimertinib treatment, drug-tolerant cells include both noncycling and cycling populations, which could evade drug pressure and lead to disease relapse (50). We further demonstrate that cotreatment of alisertib and targeted therapy led to synergistic inhibition of cycling cells, supporting the view that targeting aurora kinase could serve as a potential strategy to suppress the cycling subpopulation.

Whether combinational inhibition of cholesterol biosynthesis and aurora kinase may have synergetic effects in prevention of drug-resistance acquisition has not been assessed, to our knowledge. Through both in vitro and in vivo functional validation, we demonstrate that a triple-targeting strategy—targeting cholesterol biosynthesis, aurora kinase, and individual oncogenic drivers—can effectively eradicate almost all the cancer cells and achieve a durable tumor regression without increased systemic toxicity. These results provide proof-of-concept evidence in supporting the potential utility of the triple-targeting therapeutic strategy to suppress the onset of drug resistance toward molecular targeted therapy. Our findings also underscore a new therapeutic paradigm: by combining oncogenic targeted therapy with preemptive disruption of the early survival programs in cancer cells, we may have the chance to eliminate residual tumor cells before mutation-driven resistance is fully established. Given that both statins and alisertib are FDA-approved drugs, our triple-targeting strategy may hold significant promise in improving the efficacy of traditional molecular targeted therapy. Additionally, with the recent FDA approval of the AKT inhibitor capivasertib for breast cancer (78), studies would also be of interest to evaluate its efficacy and safety in the combination therapy of mutation-driven lung cancer.

## Methods

### Sex as a biological variable.

All animal experiments in this study were performed using female mice because they are less aggressive than male mice and, therefore, easier to work with. It is unknown whether the findings are relevant for male mice.

### Cell culture experiments.

PC9, H358, and H3122 cells were purchased from ATCC and were free of mycoplasma contamination. PC9 and H3122 cells were grown in DMEM (Hyclone) with 8% FBS (Gibco). H358 cells were grown in RPMI medium (Hyclone) with 8% FBS (Gibco). For targeted therapy, PC9 cells were treated with 10 nM gefitinib, H358 cells were treated with 10 nM sotorasib, and H3122 cells were treated with 10 nM alectinib.

### Animal studies.

All mice were housed in a specific pathogen-free environment at the Center for Excellence in Molecular Cell Science, Chinese Academy of Sciences, and treated in strict accordance with protocols approved by the Institutional Animal Care and Use Committee of the Shanghai Institutes for Biological Sciences, Chinese Academy of Sciences. For in vivo study (Supplemental Figure 1B, Figure 2, D–F, Supplemental Figure 2, G and H, Figure 5J, and Figure 6, H and I), 6-week-old nude mice were subcutaneously transplanted with 5 × 10^6^ cells until palpable tumors formed. The mice were then randomly divided into different groups (*n* = 6–9 in each group) and were treated with targeted therapy (5 mg/kg osimertinib; 25 mg/kg gefitinib; 30 mg/kg sotorasib; or 30 mg/kg alectinib) or in combination with 10 mg/kg lovastatin or 10 mg/kg alisertib by daily oral gavage. The control mice were given vehicle (1% Tween80 in double-distilled H_2_O). Tumors were monitored every 3 days and calculated by using the equation *V* = (*L* × *W* × *W*)/2, where *V* stands for volume, *L* represents length, and *W* denotes width. Mice were sacrificed and the tumors were harvested for further molecular and pathological analysis.

### Clinical specimen study.

This study included 2 distinct patient cohorts. The use of all human samples was approved by the institutional review committee of Shanghai Pulmonary Hospital, Tongji University School of Medicine. Written informed consent was obtained from all patients. In cohort 1, the tissue samples for scRNA-Seq (Figure 1, F–J) were from a prospective study (ClinicalTrials.gov identifier NCT04201756l LungMate004) (79) in which patients with stage III NSCLC harbored EGFR mutations (L858R and 19-del) received 2 to 4 cycles of neoadjuvant afatinib, as described previously (79). Primary tumor tissues were obtained via percutaneous pulmonary biopsy, bronchoscopy biopsy, or endobronchial ultrasound biopsy prior to drug administration. Fresh tumor tissues were collected immediately after surgical resection. The pretreatment samples were biopsy specimens from 5 patients with stage III NSCLC with the EGFR mutations L858R and 19-del. The posttreatment samples were surgically resected tumor specimens from the same patients after they received neoadjuvant afatinib therapy. All patients received 2 to 4 cycles of neoadjuvant afatinib therapy (each cycle lasting 4 weeks) and achieved either PR or stable disease. Radical surgery was performed within 3 weeks after discontinuing afatinib.

For cohort 2 (for immunostaining analysis; Figure 2, I and J), IHC analyses of HMGCR and SQLE were performed on a separate cohort of 41 patient samples. This cohort included 11 treatment-naive samples, 18 samples from patients with a best overall response of PR or stable disease, and 12 samples from patients with PD.

### Cell proliferation assay.

Cells were seeded in sextuplicate in 96-well plates, stained with MTT, and assessed with an Epoch multivolume spectrophotometer system (570 nm/630 nm) at indicated time points. The survival ratio of drug-treated cells was calculated by dividing the fluorescence obtained from the drug-treated cells by the fluorescence obtained from the control-treated cells (i.e., cells that received no drug treatment). Experiments were done in quadruplicate. Data are reported as mean ± SEM.

### Real-time PCR analyses.

Using Trizol reagent (Invitrogen, 15596018), total RNA was extracted from cells and reverse transcribed with a Transgene Reverse Transcription Kit (Transgene, AU341-02). cDNA was then used for real-time PCR on an LC96 System or LC480 System (Roche) with SYBR-Green Master PCR (Roche). Results were normalized to GAPDH. The sequences of primers are listed in Supplemental Table 2. Experiments were done in quadruplicate. Data are presented as mean ± SEM.

### Immunoblot assays.

Cells lysates were prepared and subjected to Western blot analysis, as described previously (80). The primary antibodies are listed in Supplemental Table 1. ImageJ was used to calculate the strength of bands. Quantification of Western blot data is shown in Supplemental Figure 7.

### Plasmid construction and virus infection.

For the p27 reporter, p27K^–^ sequenced from pCDH-EF1-mVenus-p27K^−^ (176651, Addgene) (81) was synthesized and placed into pCDH-CMV-Neo vector with mCherry sequence (82). For visualization of Akt and ERK activity, the Akt-KTR-mTurquoise2-P2A-ERK-KTR-mNeonGreen gene sequence was amplified from the vector H2A-mScarletI-P2A-Akt-KTR-mTurquoise2-P2A-ERK-KTR-mNeonGreen (129631, Addgene) (45) and was placed within pCDH-CMV-puro. Plasmids were packaged into lentiviral particles by cotransfection with packaging plasmids into HEK293T cells, and the filtered cell culture supernatant was then used to infect cells.

### Filipin staining.

For filipin staining, cells were washed 3 times with PBS and incubated with 4% paraformaldehyde (PFA) for 10 minutes at room temperature. Then cells were stained for 2 hours with 0.05 mg/mL filipin (HY-N6716, MCE) in PBS containing 10% FBS, and rinsed 3 times with PBS. The images were acquired using a Leica TCS SP8 WLL confocal microscope and were processed by LAS X (Leica).

### IHC and IF staining.

IHC staining was performed as previously described (83). In brief, tissues were fixed with 4% PFA overnight and dehydrated in ethanol, embedded in paraffin, and then sectioned (5 μm). For IHC staining, slides were deparaffinized in xylene and ethanol, and rehydrated in water. Slides were quenched in hydrogen peroxide (3%) to block endogenous peroxidase activity. Antigen retrieval was performed by heating slides in a microwave for 20 minutes in sodium citrate buffer (pH 6.0). The slides were incubated with primary antibodies at 4°C overnight and then analyzed using the SPlink Detection Kits (Biotin-Streptavidin HRP Detection Systems) following the manufacturer’s instructions. The IHC staining was blindly scored, and the IHC score was calculated as previously described (84, 85). We used the prospectively collected IHC data to generate IHC scores on a scale of 0 to 300. By integration of the data relating to the intensity and frequency of staining, the IHC score was calculated as the percentage of cells staining multiplied by positive staining strength.

For IF staining, tissues were fixed in 4% PFA overnight, then dehydrated in 30% sucrose solution overnight, embedded in OCT, sectioned (5 μm), then IF stained. Slides were incubated with primary antibodies in 1% BSA plus 0.25% Triton X-100 in PBS at 4°C overnight, incubated with secondary antibodies for 1 hour and DAPI for 10 minutes at room temperature, and then covered with coverslips for imaging. The images were acquired using a Leica TCS SP8 WLL confocal microscope and were processed by LAS X (Leica). Quantification of the KTR signal was carried out as follows: Images from 3 independent biological replicates were analyzed. For each cell, regions of interest corresponding to the nucleus (defined by DAPI staining) and the cytoplasm (manually outlined surrounding the nucleus) were delineated using ImageJ. The MFI of the AKT-KTR or ERK-KTR signals was measured within each region of interest. The cytoplasmic-to-nuclear ratio for each cell was then calculated using the following equation: ratio = mean cytoplasmic intensity/mean nuclear intensity.

### Flow cytometry.

For dormant and cycling tumor cells analysis and sorting, cells were collected after 48 hours of targeted therapy, and cell suspensions were filtered twice through 40 μm filters to obtain single cells. FACS was carried out with a BD FACSAria III sorter (BD Biosciences). Single cells were gated on the basis of their forward- and side-scatter profiles, and pulse width was used to exclude doublets. Dead cells (which appear bright on DAPI staining) were gated out. Different cell populations were isolated on the basis of expression of mCherry as dormant (mCherry^+^) and cycling (mCherry^−^) tumor cells. Postsorting analysis was performed using FlowJo (BD Biosciences).

Cell cycle analysis was performed as previously described (86). In brief, cell suspensions were collected at a concentration of 1 × 10^6^ cell/mL, and Hoechst 33342 (HY-15559, MCE) was added to a final concentration of 10 μg/mL. The cell suspensions were incubated at 37°C for 45 minutes in the dark and were mixed every 15 minutes. Then pyronin Y (HY-D0971, MCE) was directly added to cells at a final concentration of 5 μg/mL, and the cell suspensions were incubated at 37°C for another 45 minutes in the dark and mixed every 15 minutes. After incubation, supernatant was removed by centrifuging at 800*g* for 3 minutes, and cells were resuspended in PBS. The analysis was carried out with CytoFLEX LX (Beckman).

Apoptosis was assessed using an annexin V and propidium iodide double-staining kit (40305ES, YEASEN), following the manufacturer’s instructions. Briefly, cells were harvested at the indicated time points and washed twice with cold PBS. The cell pellet was resuspended in 1× binding buffer at a concentration of 1 × 10^6^ cells/mL. Annexin V-FITC (5 μL) and propidium iodide (5 μL) were added to 100 μL of the cell suspension, and the mixture was incubated for 15 minutes at room temperature in the dark. After incubation, 400 μL of 1× binding buffer was added to the samples to stop the reaction. Apoptotic cells were analyzed by flow cytometry within 1 hour of staining. Data were collected and analyzed using FlowJo (version 10.7.2).

### Bulk RNA-Seq and bioinformatic analysis.

Bulk RNA-Seq data were aligned to Hg38 genome reference by STAR (87). Gene expression was calculated according to the ENSEMBL database by using htseq-count. Read counts were used as input for the DESeq2 (version 1.36.0) package (88) for differential expression analysis. We defined differentially expressed genes (DEGs) between different conditions if the adjusted *P* value was <0.05 and fold change was >1.5. Analysis of Gene Ontology terms and Kyoto Encyclopedia of Genes and Genomes pathways of DEGs were performed using DAVID web tools (https://davidbioinformatics.nih.gov). Heatmaps were drawn using R packages (version 4.2.1) ggplot2 (version 3.4.2) and pheatmap (version 1.0.12; https://cran.r-project.org/src/contrib/Archive/pheatmap/pheatmap_1.0.12.tar.gz), in which transcripts per million values instead of raw counts are reported.

### DNB analysis.

DNB analysis (15) was performed on expression data of the cells at different periods. It has been shown that when the biological system approaches the critical state or TP during a dynamical process, expression of DNB genes simultaneously satisfies 3 generic properties: (1) the average Pearson’s correlation coefficient (PCC_in_) of DNB genes as a group drastically increases; (2) the average Pearson’s correlation coefficient (PCC_out_) of DNB genes between this group and any others drastically decreases; and (3) the average SD (SD_in_) of DNB genes in this group drastically increases (15). These 3 generic properties could be combined to construct a DNB composite index (CI), as follows: CI = SD_in_ (PCC_in_/PCC_out_), to estimate the critical state or TP during a dynamical process based on the cells of each period. When the CI at a period reaches the highest value, the state of the samples at this sliding window is considered as the critical state or TP. The corresponding DNB genes are considered the leading molecules of this critical transition.

### scRNA-Seq data preprocessing.

scRNA-Seq data (Singleron Biotechnologies) were generated using CeleScope (https://github.com/singleron-RD/CeleScope) with default parameters. Then the gene expression matrices were used for downstream analysis by the Seurat R package (version 4.4) (89). In the preprocessing stage, we applied a gene-count filter to eliminate low-quality libraries. Specifically, any cell with a total number of detected genes below 250 (nFeature_RNA < 250), having unique counts exceeding 60,000 or below 500, and expressing mitochondrial RNA exceeding 30% was filtered out. We used the fastMNN function to remove the batch effect based on the mutual nearest neighbors (MNN) (90). Then the NormalizeData and ScaleData functions were applied to normalize the expression matrices, and the FindVariable function was applied to select the top 2,000 variable genes and perform principal component analysis. The first 10 principal components and resolution 0.1 were used with FindClusters function to generate cell clusters.

### Identification of malignant cells.

To identify malignant cells from epithelial cells, we used inferCNVpy (91) to estimate the copy number variations (CNVs). The stromal cells were used as normal reference. Then we used default parameters to calculate the CNV score for each gene per cell. We centered the relative expression values to 1 and used a 1.5 SD of the residual normalized expression values as the ceiling and floor for visualization using R package pheatmap to determine the malignant cells (92). We further clustered malignant cells with the resolution parameter of 0.1 and first 10 principal components to generate cell subtype.

### Calculate cholesterol pathway score.

To evaluate the cholesterol pathway, we applied the HALLMARK datasets from the MSigDb (Molecular Signatures Database; https://www.gsea-msigdb.org/gsea/msigdb) to calculate cholesterol based on the AUCell method with default parameters (93).

### Trajectory analysis.

We applied Monocle 2 to determine the lineage differentiation of cell subtypes with a potential developmental relationship to malignant cells. The FindVariableFeatures function was used to select top 2,000 high-variable genes to order cells. We used the DDRTree method to learn tree-like trajectories (21).

### Differential expression analysis.

Differential expression analysis comparing cells from treatment and control groups was performed using the FindMarkers function with the parameter “min.pct=0.1, logfc.threshold=0.25.” Then, we used the R package clusterProfiler to do the Gene Ontology enrichment with HALLMARK pathway. We also applied gene set variation analysis (GSVA) using standard settings, as implemented in the GSVA R package to estimate the activity pathway in each cell cluster (94).

### ATAC-Seq and bioinformatics analysis.

Reads from ATAC-Seq datasets were mapped to the Hg38 reference genome by Bowtie2 (version 2.3.1) (95). Multiple mapped reads and PCR duplicates of ATAC-Seq were removed with the sambamba (version 0.6.6) (96) markdup command. Peaks were called by MACS2 (97) with a *P* value <0.01. MACS2 was then used for peak calling. The reads, after removing duplicates, were normalized by bamCoverage (version 1.5.11) (98) to calculate the reads per genome coverage. The WashU Epigenome Browser (http://epigenomegateway.wustl.edu/browser/) and R packages ggplot2 (version 3.4.2) and heatmaply (version 1.4.0) (99) were used to visualize the normalized data.

### Statistics.

Statistical analysis was performed using GraphPad Prism 7 software. An unpaired 2-tailed *t* test was used for comparison of 2 groups, and 1-way ANOVA with Dunnett’s multiple comparisons test was used for comparison of 3 or more groups. Data are presented as mean ± SEM. Statistical significance was defined as *P* < 0.05. Levels of significance are indicated in the figure legends.

### Study approval.

This study was approved by the Ethics Committee of Shanghai Pulmonary Hospital. Written informed consent was obtained from all patients.

### Data and materials availability.

All plasmids generated in this study are available upon request, with a completed Materials Transfer Agreement, from the corresponding author.

The RNA-Seq data of EGFR-mutant NSCLC cell line PC9 reported here have been deposited in OMIX, China National Center for Bioinformation/Beijing Institute of Genomics, Chinese Academy of Sciences (https://ngdc.cncb.ac.cn/omix; accession no. OMIX007899), as have the ATAC-Seq data of EGFR-mutant NSCLC cell line PC9 (https://ngdc.cncb.ac.cn/omix; accession no. OMIX007918). The scRNA-Seq data generated in this study have been deposited in the Genome Sequence Archive database of the National Genomics Data Center (https://bigd.big.ac.cn/) under BioProject accession PRJCA032501. All processed scRNA-Seq data contained in the manuscript and in its supplemental information are available upon request. Values for all data points in graphs are reported in the Supporting Data Values file.

## Author contributions

Y Zhao and HJ conceptualized the study. LC devised the methodology. Y Zhao, Y Zhou, and LC conducted the investigation. Y Zhao and Y Zhou contributed to validation. LP, GT, and ST conducted the formal analysis. HYH and LZ contributed resources. LC curated the study data. LC, LZ, LH, and HJ supervised the study. LC, LH, and HJ acquired funds for the study. LH and HJ served as project administrators. Y Zhao wrote the original draft of the manuscript. Y Zhao, Y Zhou, LH, and JI reviewed and edited the manuscript.

## Conflict of interest

The authors have declared that no conflict of interest exists.

## Funding support

- National Key Research and Development Program of China (grants 2022YFA1103900 and 2020YFA0803300 to HJ; and 2022YFA1004800, 2025YFF1207900, and 2025YFC3409300 to LC).
- National Natural Science Foundation of China (grants 82341002, 32293192, and 82030083 to HJ; 82473426 and 82173340 to LH; T2350003, 12131020, 42450084, 42450135, 12326614, 12426310, and T2542018 to LC; and 82303575 to ST).
- Innovative research team of high-level local universities in Shanghai (grant SSMU-ZLCX20180500 to HJ).
- Science and Technology Commission of Shanghai Municipality (grant 23JS1401300 to LC).
- Zhejiang Province Vanguard Goose-Leading Initiative (grant 2025C01114 to LC).
- Shenzhen Medical Research Fund (grants E250200620 and E250200621 to LC).

## Supplementary Material

Supplemental data

Unedited blot and gel images

Supplemental tables 1-2

Supporting data values

## Version 1 04/15/2026

Electronic publication
