# Supplementary material for: Therapy-induced cholesterol biosynthesis drives lung cancer dormancy and drug resistance
Source: J Clin Invest. 2026 Apr 15;136(8):e191735. doi: 10.1172/JCI191735 (PMC13078878; doi:10.1172/JCI191735)
Supplement: Supplemental data [file jci-136-191735-s042.pdf]

Supplementary Data for

Therapy-induced cholesterol biosynthesis drives lung cancer dormancy and drug resistance

Yikai Zhao[1,10], Yijia Zhou[2,10], Linnuo Pan[1,10], Geng G. Tian[3], Hsin-Yi Huang[1], Shijie Tang[1], Ming Lu[4], Zhangsen Zhou[4], Peng Zhang[5], Luonan Chen[6,9], Lele Zhang[5,*], Liang Hu[7,8,*], Hongbin Ji[1,2,6,7,8*]

Supplementary figures and legends

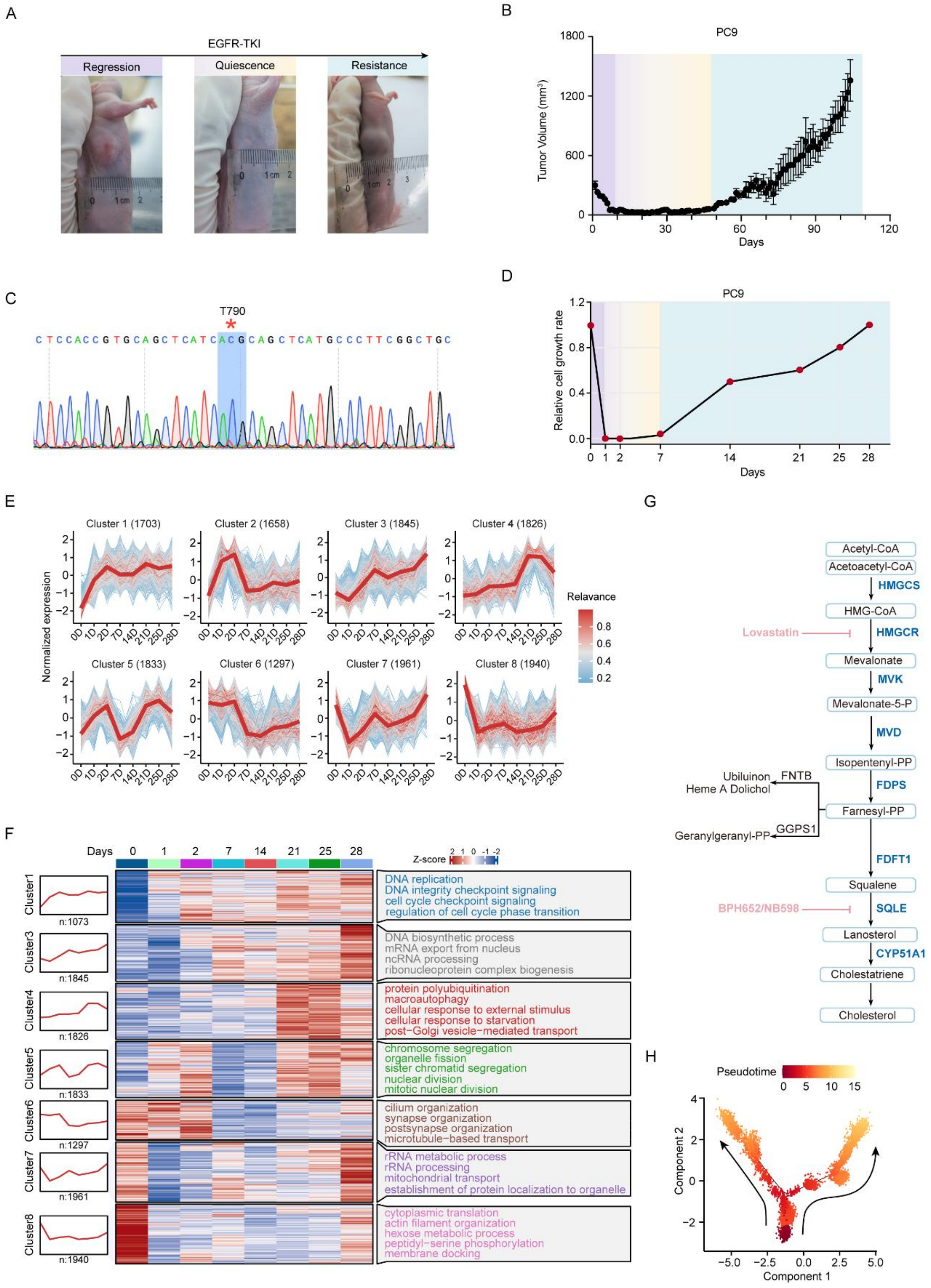

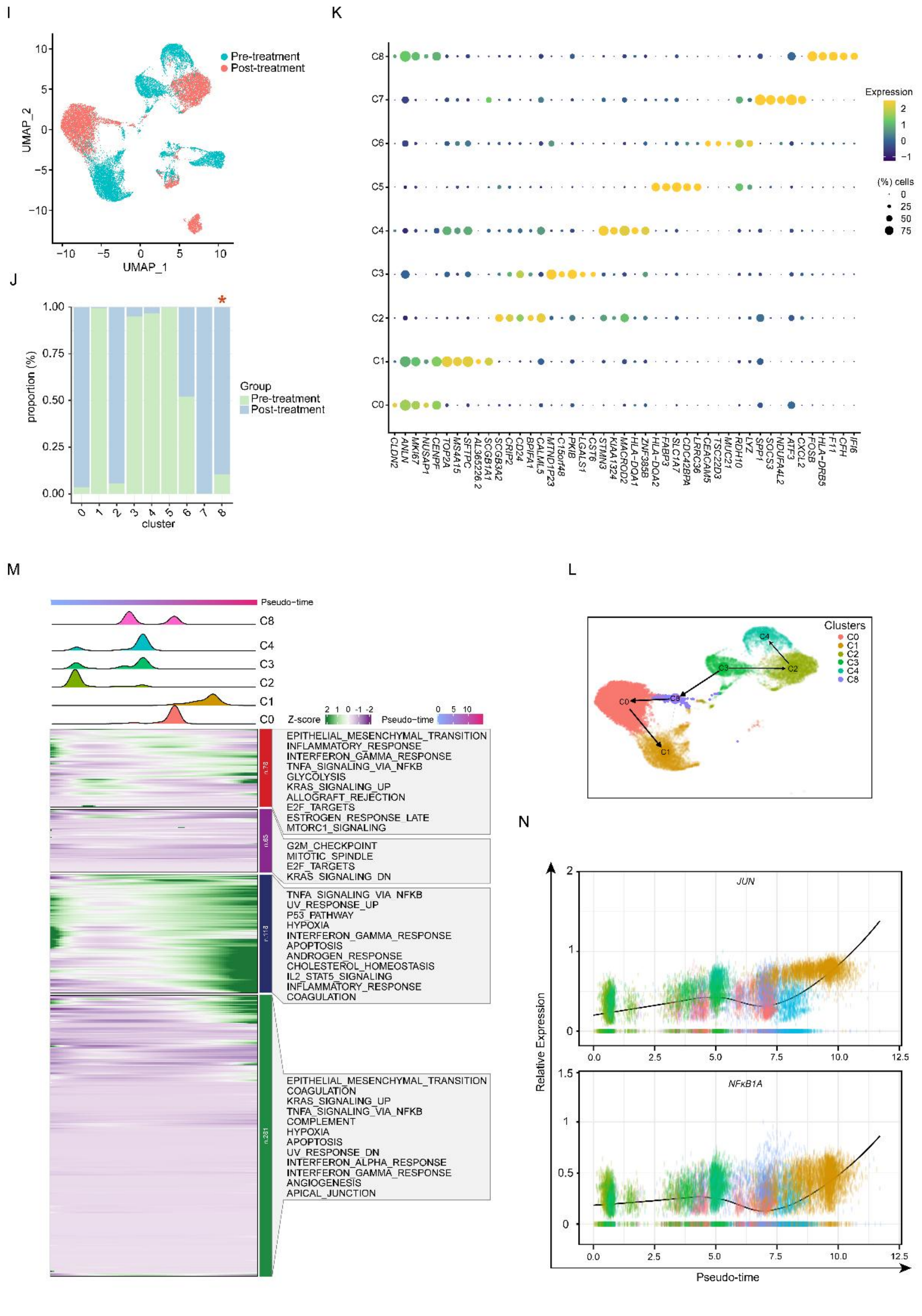

Figure S1, related to Figure 1. Targeted therapy activates cholesterol biosynthesis

(A) Representative image of PC9 xenografts illustrating the transition from a drug-sensitive state to a drug-resistant state, showing three distinct phases: tumor regression,

dormancy, and drug resistance. **(B)** Tumor growth curve of PC9 xenografts treated daily with Gefitinib by oral gavage. Tumor volumes were measured every 3 days (n = 5). **(C)** DNA sequencing analysis of PC9 xenografts after 104 days of Gefitinib treatment, highlighting sequencing signals around the T790 mutation site, with no mutation detected. **(D)** Cell growth curve of PC9 cells continuously treated with Gefitinib (10 nM). **(E)** Gene trends across the development of acquired drug resistance of RNA-seq analyses. Solid lines represent the mean gene trends for each cluster respectively. **(F)** Heatmap showing averaged signature enrichment scores of cluster 1,3,4,5,6,7,8 in different time. **(G)** Schematic illustration of the cholesterol synthesis pathway. Lovastatin targets HMGCR, and BPH652 and NB598 target SQLE in the pathway. **(H)** Pseudotime trajectory analysis of tumor cells with Monocle 2. Black arrows indicated the two predicted routes of tumor cells. **(I)** Comparative UMAP visualization of pre- versus post-treatment cellular states in 5 paired lung cancer samples (scRNA-seq). **(J)** Proportion of cells in each cluster between Pre- and Post-treatment group. **(K)** Dot plot showing the marker genes expression across all clusters (C0-C8). Dot diameter indicates the proportion of cells expressing a given gene; color indicates the relative expression level. **(L)** Upper Panel: Density plots depict the distribution of key tumor cell subpopulations (C0, C1, C2, C3, C4, C8) along the pseudotime trajectory 2, revealing their dynamic changes. Lower Panel: A heatmap quantifies the activation of hallmark biological processes across these subpopulations over pseudotime trajectory 2, with color intensity representing the gene expression Z-score. **(M)** PAGE analysis illustrating the pseudotime trajectory 2 direction of tumor cell subpopulations. **(N)** Relative genes expression across the pseudotime trajectory 2.

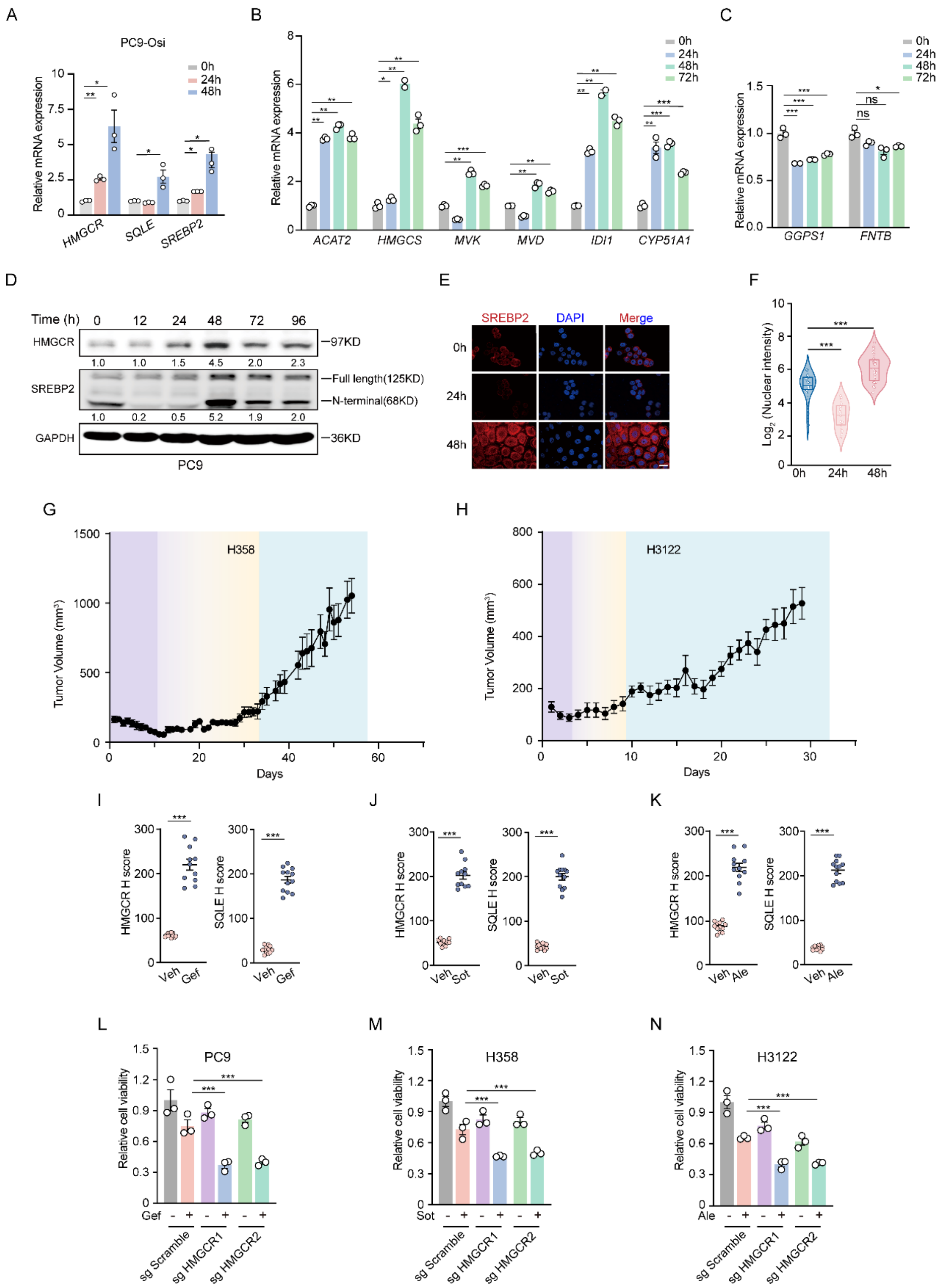
A
PC9-Osi
Relative mRNA expression
HMGCR
SQLE
SREBP2
0h
24h
48h
B
Relative mRNA expression
ACAT2
HMGCS
MVK
MVD
IDI1
CYP51A1
0h
24h
48h
72h
C
Relative mRNA expression
GGPS1
FNTB
ns
D
Time (h)
HMGCR
SREBP2
GAPDH
—97KD
—Full length(125KD)
—N-terminal(68KD)
—36KD
PC9
E
SREBP2
DAPI
Merge
F
Log2 (Nuclear intensity)
G
H358
Tumor Volume (mm3)
Days
H
H3122
Tumor Volume (mm3)
Days
I
HMGCR H score
SQLE H score
Veh
Gef
J
Veh
Sot
K
Veh
Ale
L
PC9
Relative cell viability
sg Scramble
sg HMGCR1
sg HMGCR2
M
H358
N
H3122

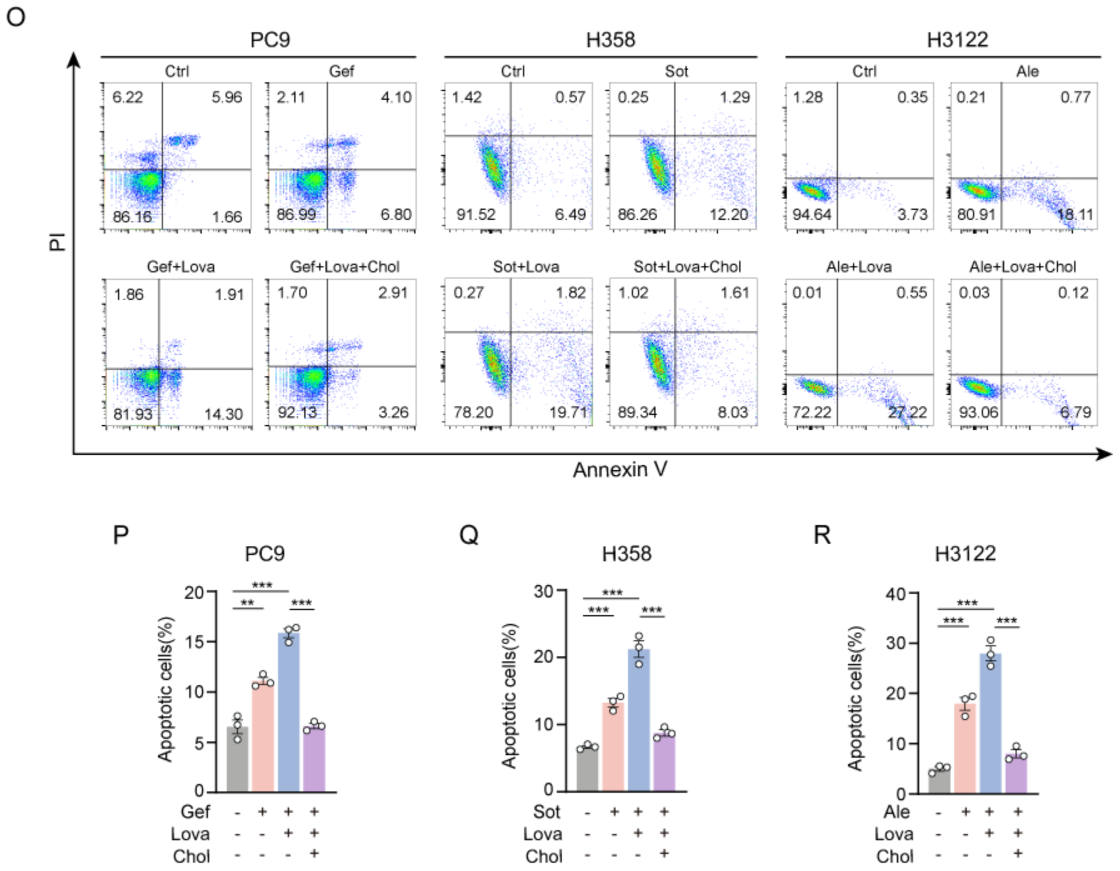

Figure S2, related to Figure 2. Cholesterol promotes lung cancer cell survival during targeted therapy

(A) Real-time PCR detection of mRNA levels of *HMGCR*, *SQLE* and *SREBP2* in PC9 cells treated with Osimertinib for the indicated times. **(B-C)** Real-time PCR detection of mRNA levels of enzymes involved in the cholesterol synthesis pathway (B) or branch pathway (C) in PC9 cells treated with Gefitinib for the indicated times. **(D)** Western blot analysis of HMGCR and SREBP2 expression in PC9 cells treated with Gefitinib for the indicated times. **(E-F)** Representative immunofluorescence staining of SREBP2 (E) and quantification (F) in PC9 cells treated with Gefitinib for the indicated times. Scale bar, 10 μm. **(G-H)** Tumor growth curve of H358 (G) or H3122 (H) xenografts in mice treated daily with Sotorasib or Alectinib by oral gavage. Tumor volumes were measured every 3 days (n = 5 each group). **(I-K)** H score for HMGCR and SQLE in PC9 (I), H358 (J), H3122 (K) xenografts after targeted therapies. Each point represents quantification from one representative immunohistochemistry (IHC)

image. **(L-N)** Relative viability of PC9 (L), H358 (M), and H3122 cells (N) with or without HMGCR knockout treated with targeted therapies for 48 h. **(O)** Representative flow cytometry dot plots of Annexin V/PI staining in PC9 (left), H358 (middle), and H3122 cells (right) treated with targeted therapies, or in combination with Lovastatin (5 μM), or in combination with Lovastatin (5 μM) and MβCD-coated cholesterol (10 μg/ml) for 48 h. **(P-R)** Quantitative analysis of apoptosis rates (early + late apoptosis) in (O). Data in (A-E, L-R) represent one experiment of three independent experiments. For western blot analysis, GAPDH served as the internal control. *P < 0.05, **P < 0.01, ***P < 0.001 by one-way ANOVA with Dunnett's multiple comparisons test in panel (A-C, F, I-Q, S-U), ns: not significant. Data are represented as mean ± SEM.

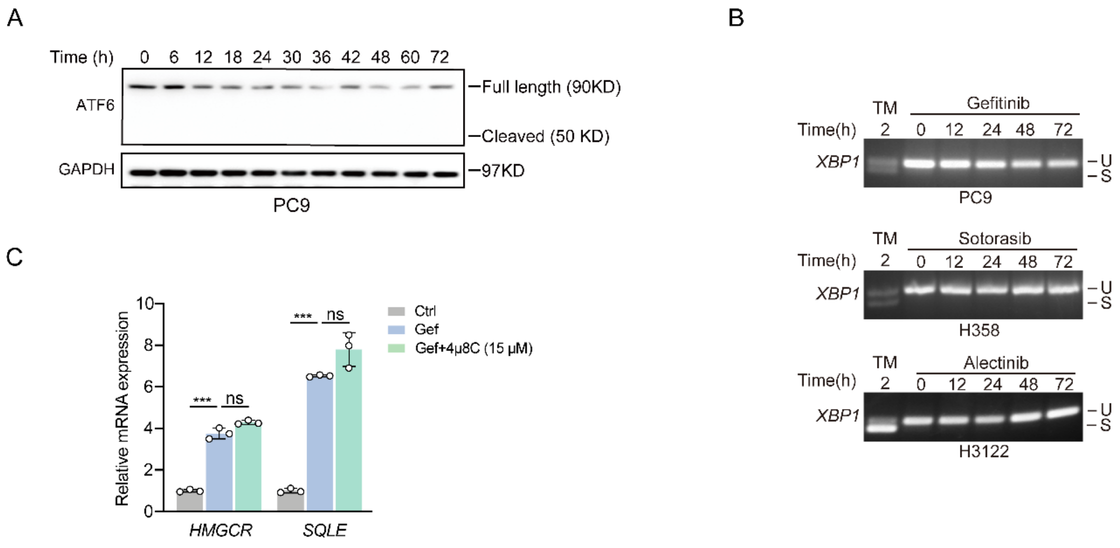

Figure S3, related to Figure 3. PERK-eIF2α signaling contributes to cholesterol biosynthesis

(A) Western blot analysis of ATF6 expression in PC9 cells treated with Gefitinib for the indicated times. **(B)** PCR analysis of *XBP1* splicing in PC9, H358, H3122 cells treated with targeted therapy for the indicated times. U, unspliced. S, spliced. Treatment of tunicamycin (TM, 1 μg/ml) for 2 hours was served as a positive control. Results are representative of three independent experiments with similar results. **(C)** Real-time PCR detection of HMGCR, SQLE mRNA levels in PC9 cells treated with Gefitinib

alone or in combination with 4μ8C (15 μM) for 48 h. Real-time PCR data in (C) represent one experiment of three independent experiments. ***P < 0.01*, ****P < 0.001* by one-way ANOVA with Dunnett's multiple comparisons test in panel (C). Data are represented as mean ± SEM.

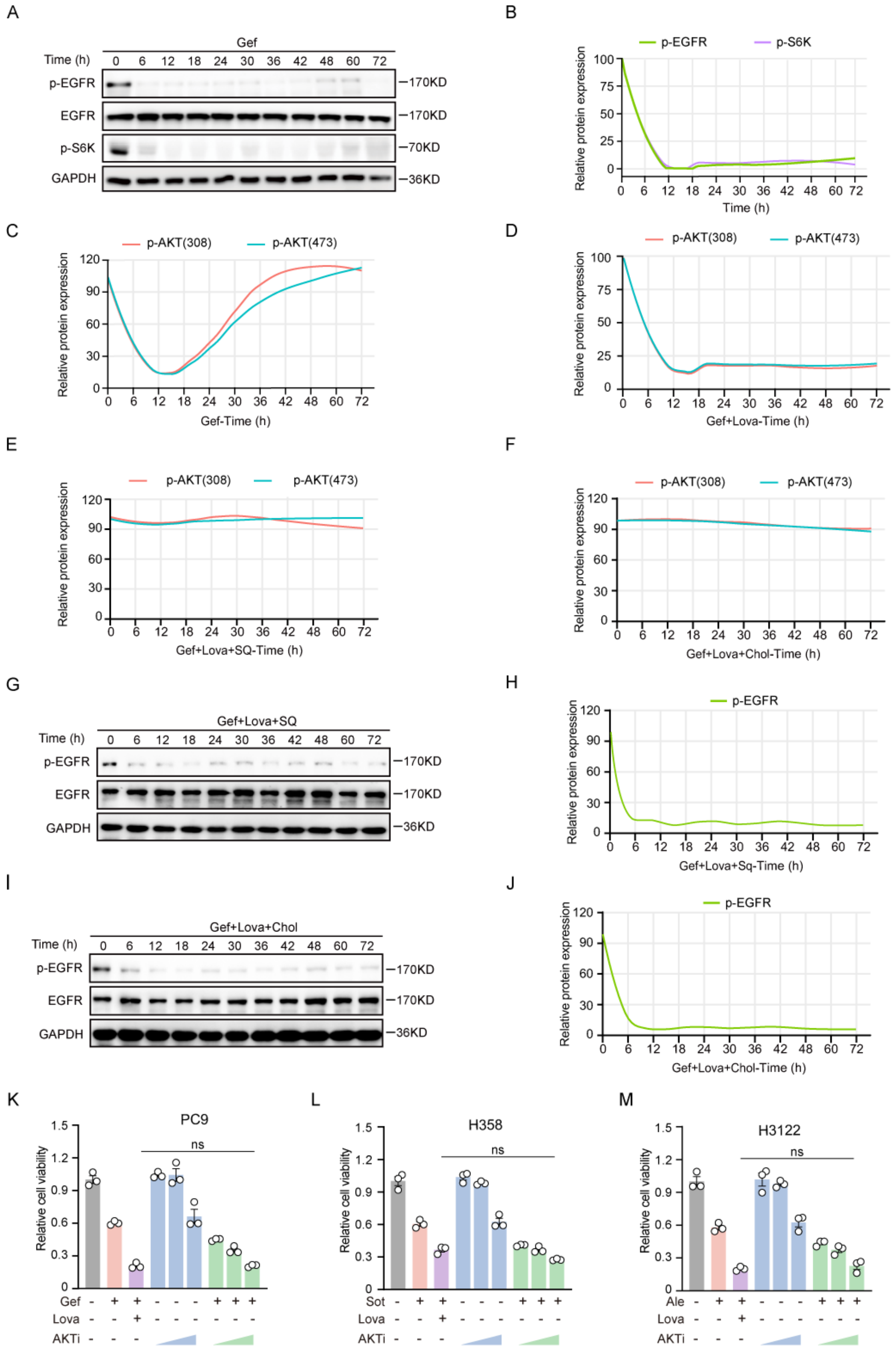

Figure S4, related to Figure 4. Cholesterol biosynthesis promotes AKT activation

(A) Western blot analysis of p-EGFR and p-S6K in PC9 cells treated with Gefitinib for the indicated times. Figure 4I and Supplementary Figure 4A were derived from the same batch of samples. They share a common GAPDH loading control to confirm equal protein loading across all lanes. **(B)** Quantification of p-EGFR and p-S6K in panel (A) **(C)** Quantification of p-AKT Ser308 and Ser473 in Figure 4 panel (I). **(D)** Quantification of p-AKT Ser308 and Ser473 in Figure 4 panel (J). **(E)** Quantification of p-AKT Ser308 and Ser473 in Figure 4 panel (K). **(F)** Quantification of p-AKT Ser308 and Ser473 in Figure 4 panel (L). **(G)** Western blot analysis of p-EGFR in PC9 cells treated with Gefitinib in combination with Lovastatin (5 μM) and Squalene (0.5 μM) for the indicated times. **(H)** Quantification of p-EGFR in panel (G). **(I)** Western blot analysis of p-EGFR in PC9 cells treated with Gefitinib in combination with Lovastatin (5 μM) and MβCD-coated cholesterol (10 μg/ml) for the indicated times. **(J)** Quantification of p-EGFR in panel (I). **(K-M)** Relative viability of PC9 (K), H358 (L), and H3122 (M) cells treated with targeted therapy alone, or AKT inhibitor VIII (AKTi; 1, 2, 4 μM) alone, or targeted therapy combined with Lovastatin (5 μM), or targeted therapy combined with AKTi (1, 2, 4 μM) for 48h. For western blot analysis, GAPDH was used as an internal control.

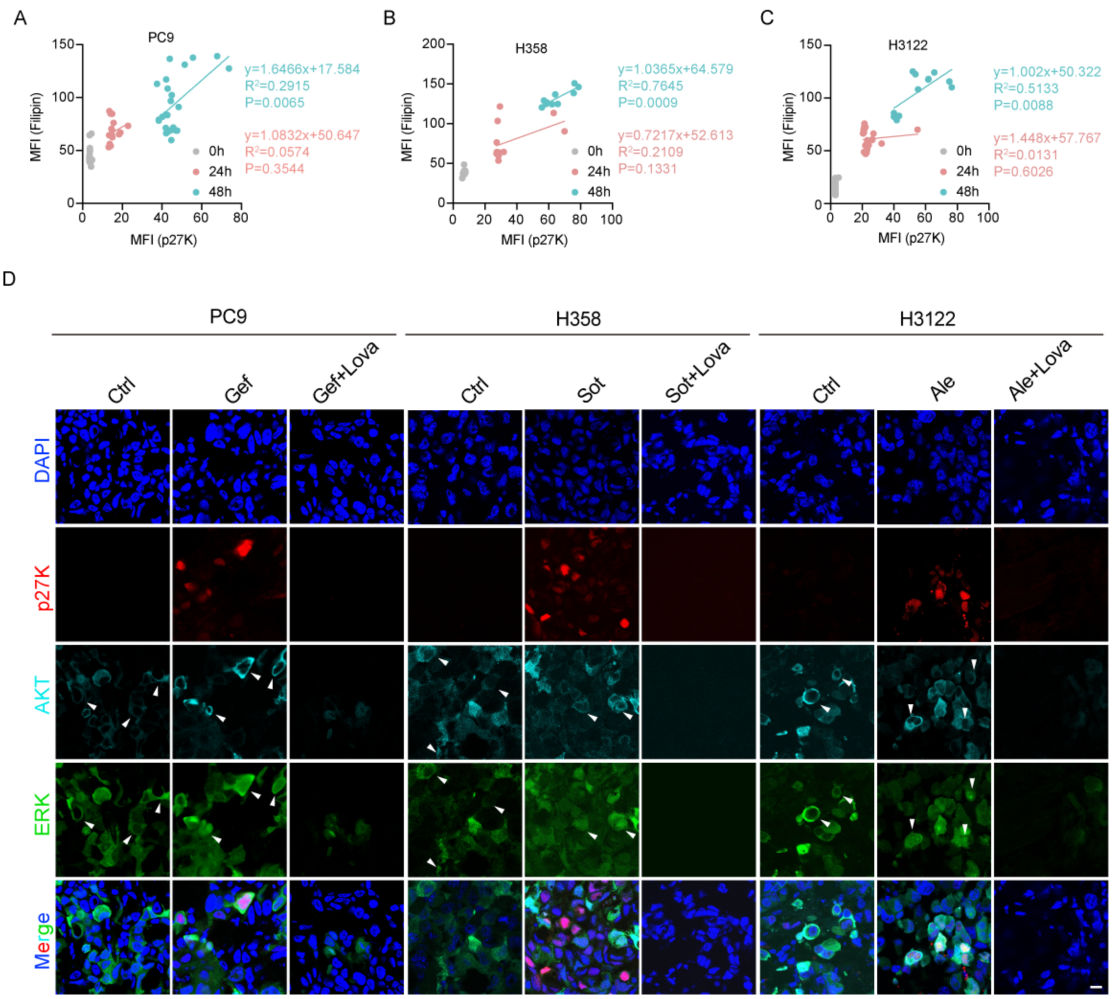

Figure S5, related to Figure 5. Dormant cancer cells exhibit high levels of cholesterol and AKT activation

(A-C) Scatter plots showing the correlation between filipin fluorescence intensity and p27K expression in PC9 (A), H358 (B) and H3122 (C) treated with targeted therapies for the indicated times. **(D)** Representative immunofluorescence images of xenograft tissue expressing p27K and AKT/ERK-KTR. AKT-KTR and ERK-KTR are shown in cyan and green, respectively, while p27K is in red, and nuclei are stained with DAPI (blue). Scale bar, 10 μm.

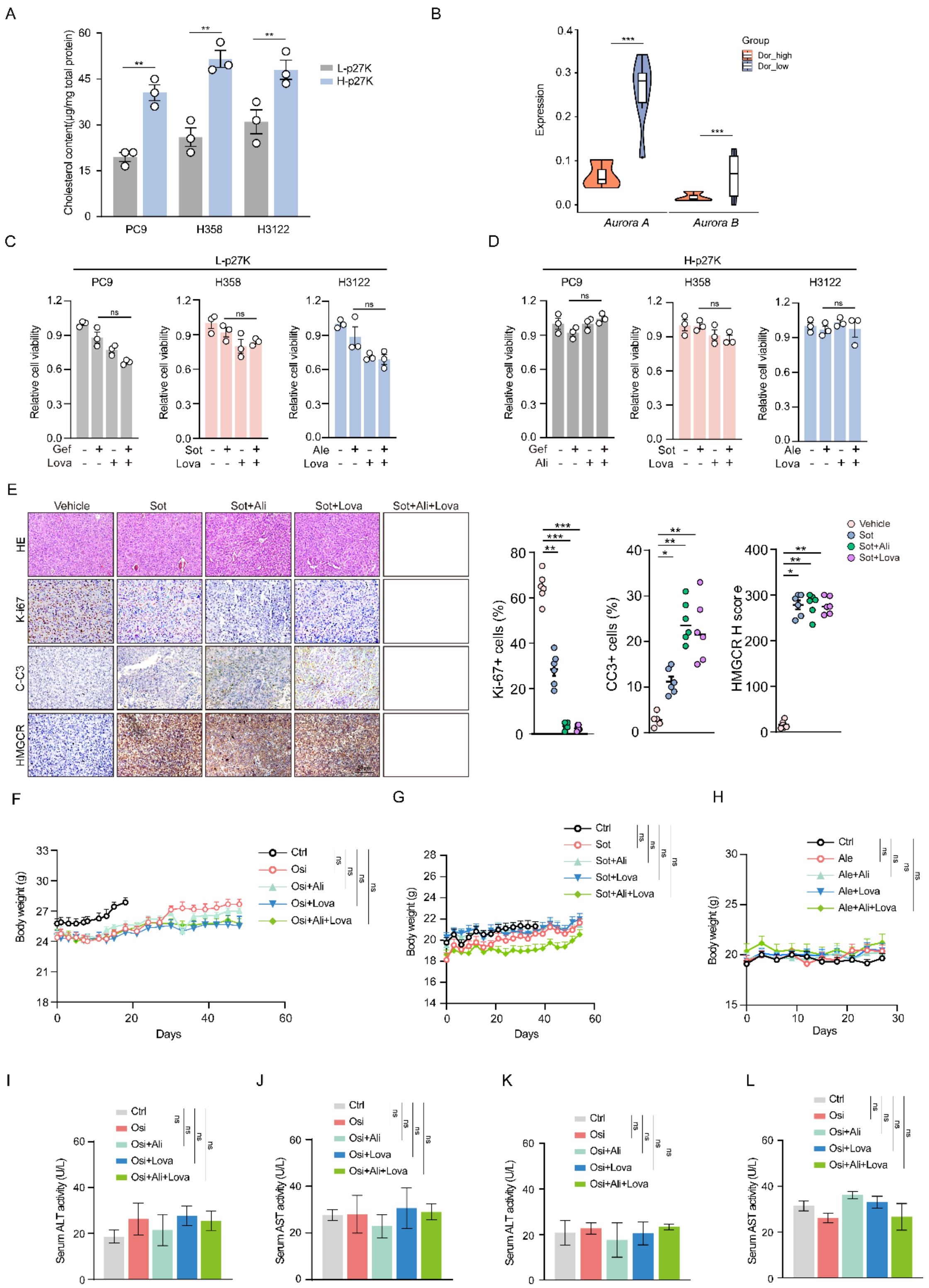

Figure S6, related to Figure 6. Combination of targeted therapy with Lovastatin and Alisertib show synergistic anti-tumor activity

(A) The total cholesterol content of H-p27K and L-p27K subpopulations in PC9, H358

and H3122 cells treated with targeted therapies for 48 h. **(B)** Violin Plots showing the relative expression of *Aurora A* and *B* in cancer cells with low and high dormancy score in the scRNA-seq data from post-treatment samples used in the study. **(C-D)** Relative viability of L-p27K (C) or H-p27K (D) subpopulations in PC9, H358 and H3122 cells treated with targeted therapies, or in combination with Lovastatin (5 μM) or Alisertib (5 nM) for 48 h. **(E)** Representative H&E and IHC staining for Ki-67, Cleaved Caspase-3 (CC3), and HMGCR in tumors from mice treated with vehicle, Sotorasib, or in combination with Lovastatin, Alisertib, or both (Left); Quantitative analysis of Ki-67-positive cells, CC3-positive cells, and HMGCR H-Score (Right). Each point represents quantification from one representative IHC image. Scale bar, 50 μm. **(F-H)** Body weight of PC9 (F), H358 (G) and H3122 (H) xenograft-bearing mice treated with vehicle, targeted therapies, or in combination with Lovastatin, Alisertib, or both. **(I-L)** ALT (I, K) and AST (J, L) levels in serum of wild type (non-tumor-bearing) nude mice (I-J) and PC9 xenograft-bearing mice (K-L) treated with vehicle, targeted therapies, or in combination with Lovastatin, Alisertib, or both. *$P < 0.05$, **$P < 0.01$, ***$P < 0.001$ by one-way ANOVA with Dunnett's multiple comparisons test in panel (C-L), two-tailed unpaired Student's t test (A, B), ns: not significant. Data are represented as mean ± SEM.

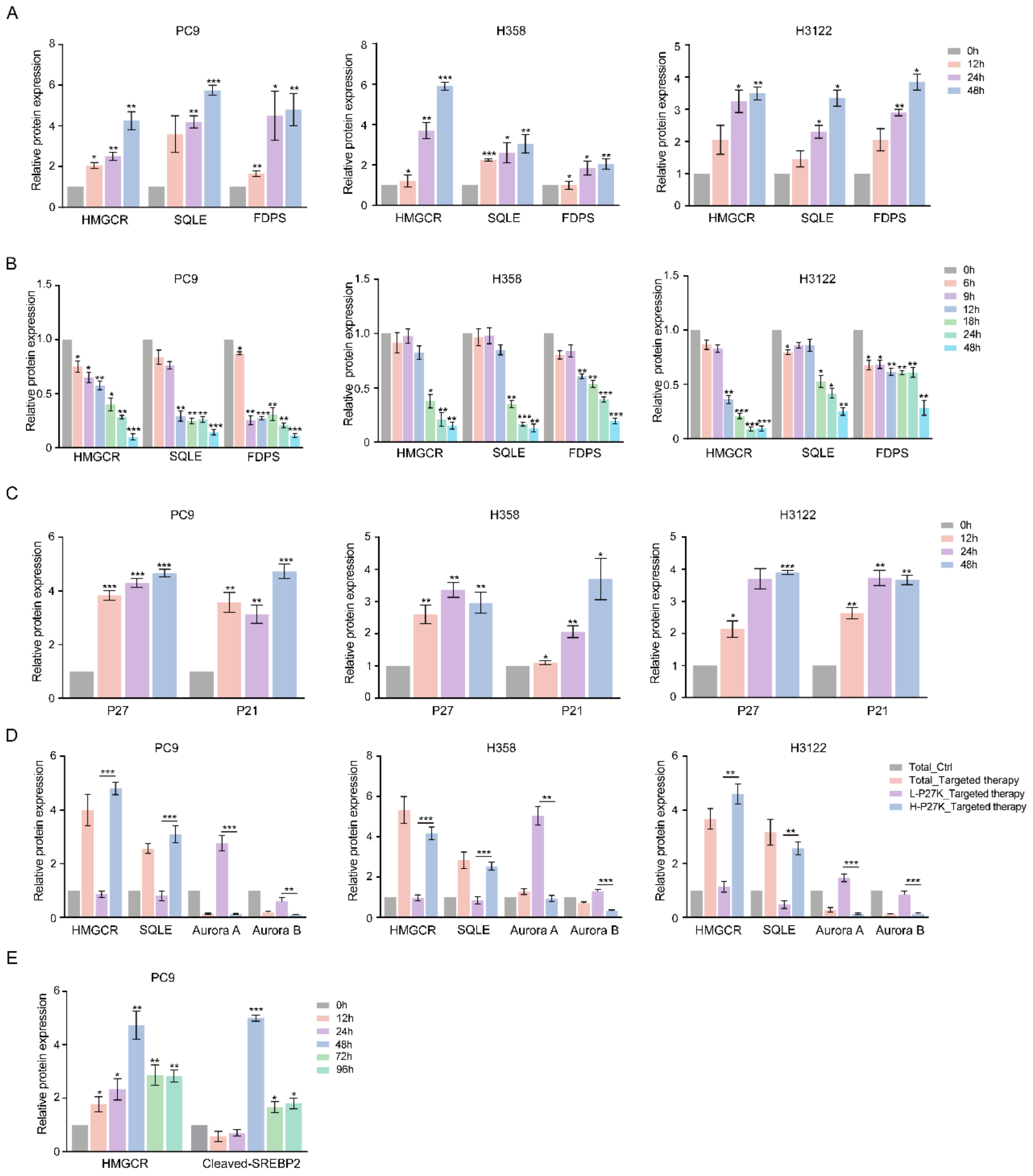

Figure S7, Quantification of Western blot analysis.

(A) Quantification of HMGCR, SQLE and FDPS protein levels in PC9, H358 and H3122 cells as treated in Figure 2B. (**B)** Quantification of HMGCR, SQLE and FDPS protein levels in PC9, H358 and H3122 cells as treated in Figure 3E. (**C)** Quantification of P27 and P21 protein levels in PC9, H358 and H3122 cells as treated in Figure 5A. (**D)** Quantification of HMGCR, SQLE, Aurora A and B protein levels in PC9, H358 and H3122 cells as treated in Figure 6D. (**E)** Quantification of HMGCR and cleaved

SREBP2 protein levels in PC9 cells as treated in Figure S2D. *$P < 0.05$, **$P < 0.01$, ***$P < 0.001$ by one-way ANOVA with Dunnett's multiple comparisons test, Data are represented as mean ± SEM.
