# Supplementary material for: Therapy-induced cholesterol biosynthesis drives lung cancer dormancy and drug resistance
Source: J Clin Invest. 2026 Apr 15;136(8):e191735. doi: 10.1172/JCI191735 (PMC13078878; doi:10.1172/JCI191735)
Supplement: Unedited blot and gel images [file jci-136-191735-s043.pdf]

Gel raw data

Figure 2B

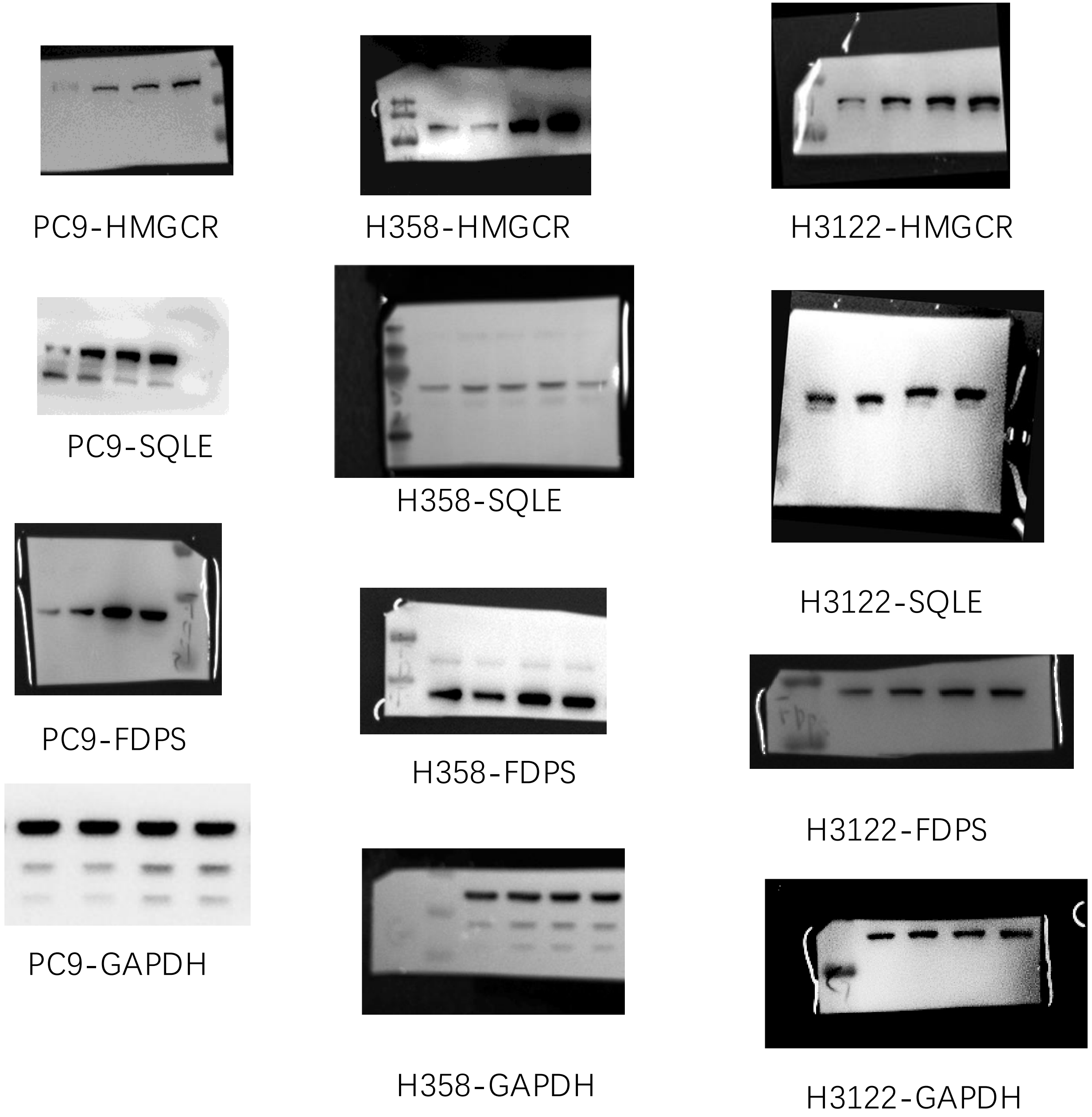
PC9-HMGCR
H358-HMGCR
H3122-HMGCR
PC9-SQLE
H358-SQLE
H3122-SQLE
PC9-FDPS
H358-FDPS
H3122-FDPS
PC9-GAPDH
H358-GAPDH
H3122-GAPDH

Figure 3B

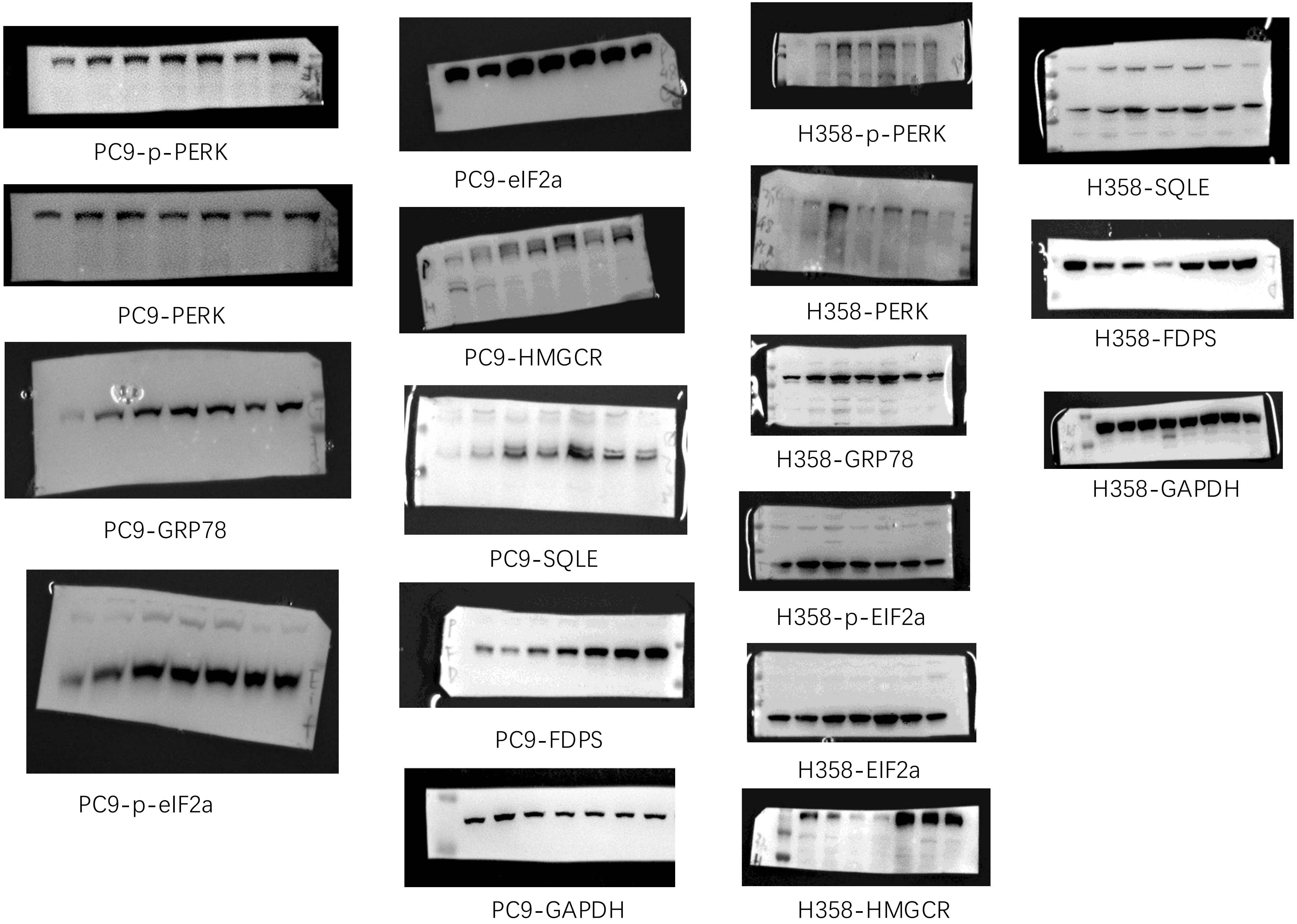
PC9-p-PERK
PC9-PERK
PC9-GRP78
PC9-p-eIF2a
PC9-eIF2a
PC9-HMGCR
PC9-SQLE
PC9-FDPS
PC9-GAPDH
H358-p-PERK
H358-PERK
H358-GRP78
H358-p-EIF2a
H358-EIF2a
H358-HMGCR
H358-SQLE
H358-FDPS
H358-GAPDH

Figure 3B

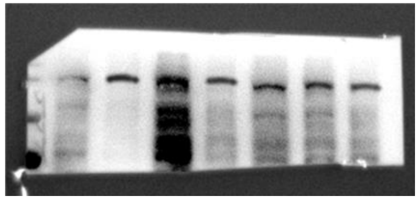

H3122-p-PERK

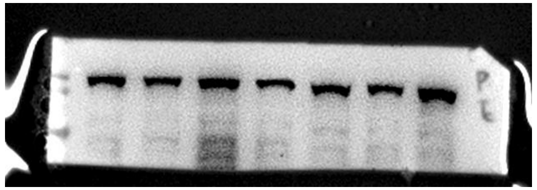

H3122-PERK

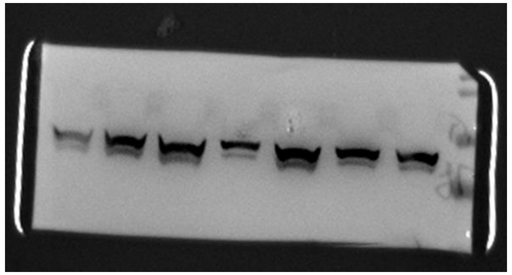

H3122-GRP78

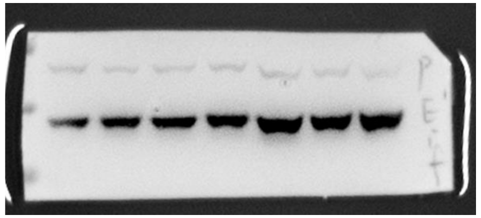

H3122-p-EIF2a

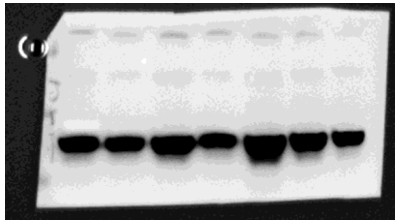

H3122-EIF2a

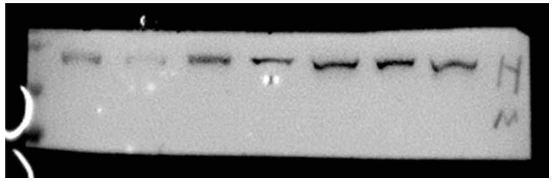

H3122-HMGCR

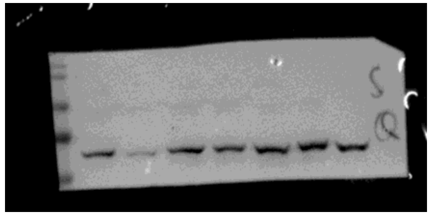

H3122-SQLE

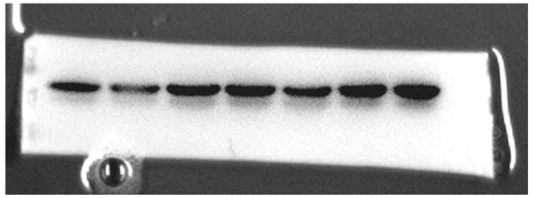

H3122-FDPS

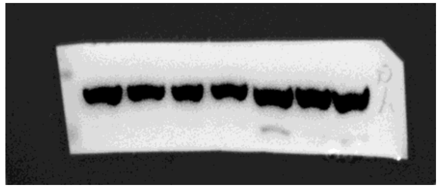

H3122-GAPDH

Figure 3E

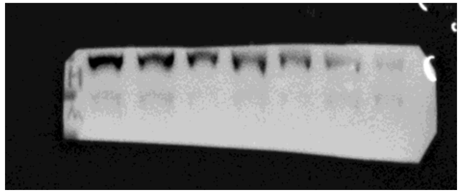
PC9-HMGCR

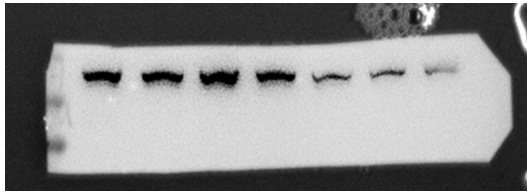
H358-HMGCR

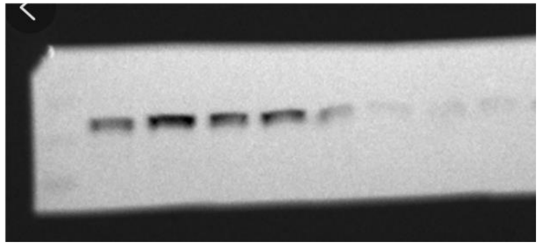
H3122-HMGCR

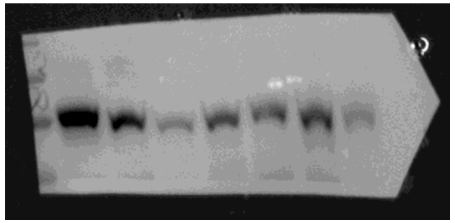
PC9-SQLE

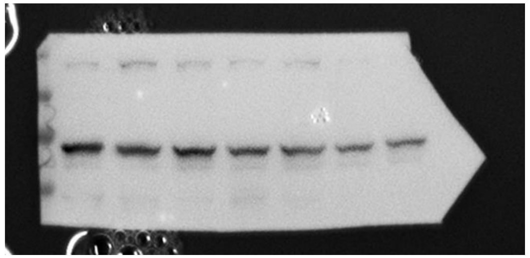
H358-SQLE

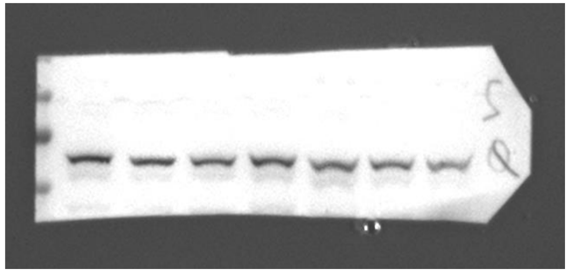
H3122-SQLE

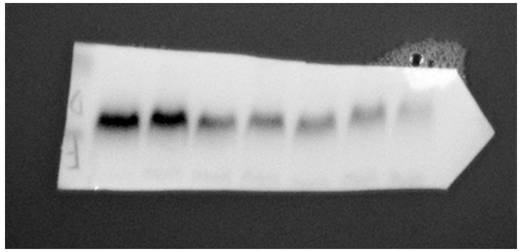
PC9-FDPS

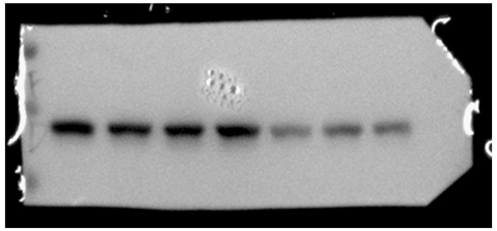
H358-FDPS

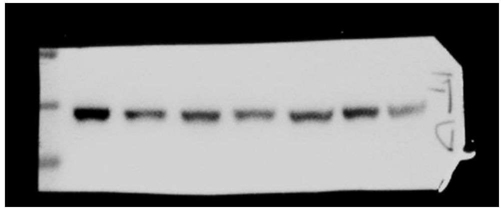
H3122-FDPS

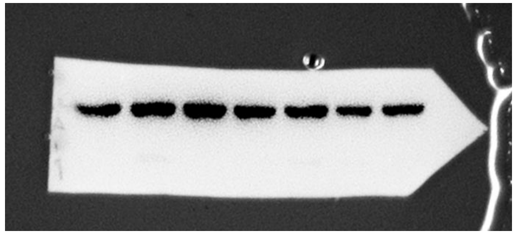
PC9-GAPDH

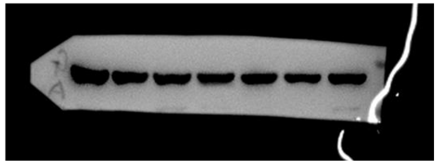
H358-GAPDH

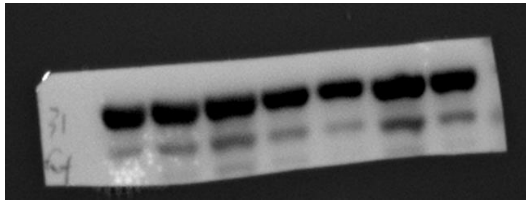
H3122-GAPDH

Figure 4I

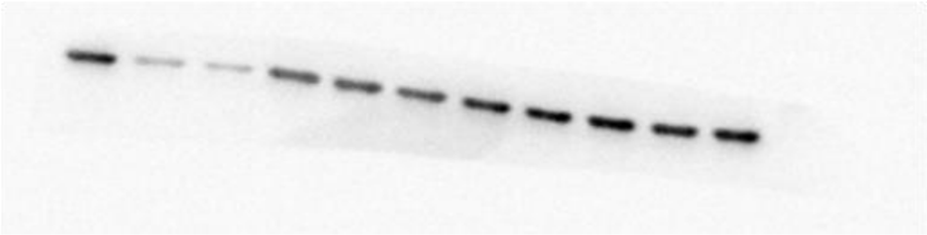

Gef p-AKT（308)

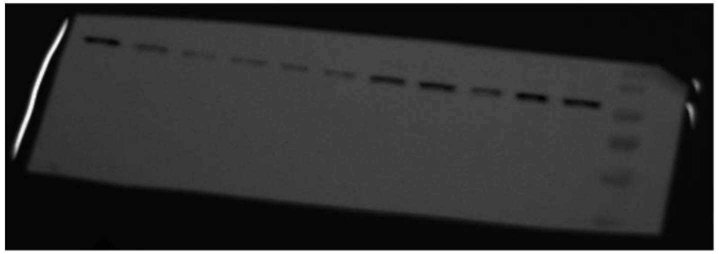

Gef p-AKT（473)

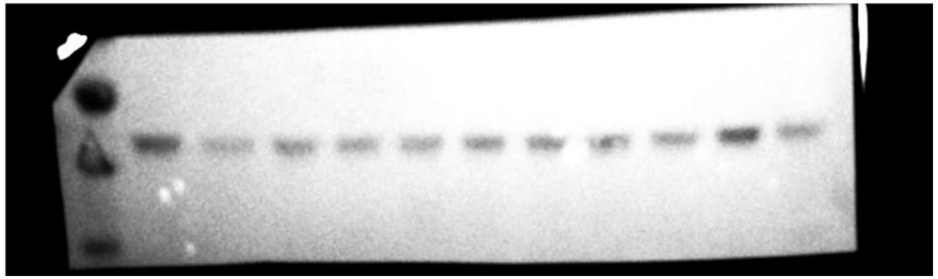

Gef AKT

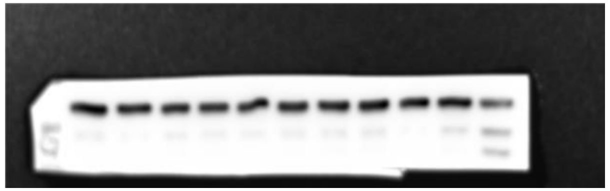

Gef GAPDH

Figure 4J

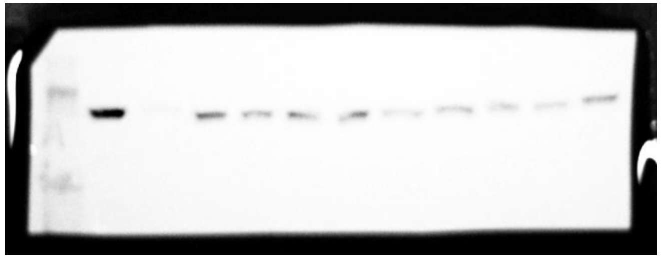

Gef+Lova p-AKT（308)

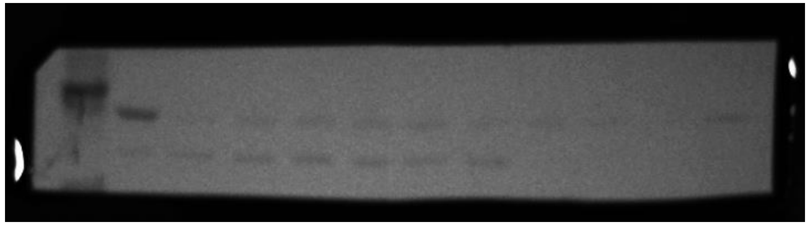

Gef+Lova p-AKT（473)

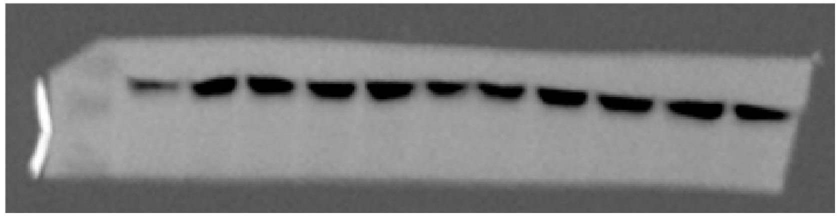

Gef+Lova AKT

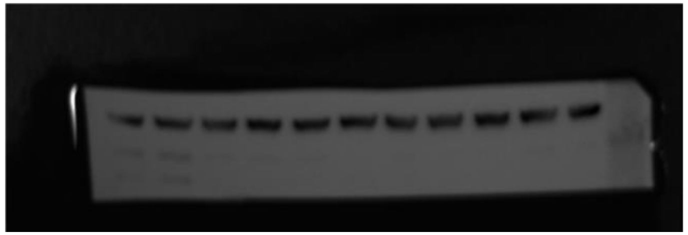

Gef+Lova GAPDH

Figure 4K

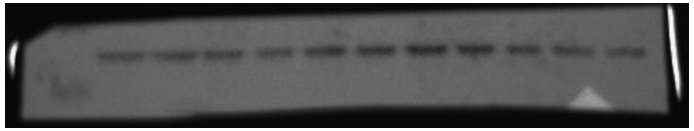

Gef+Lova+SQ p-AKT(308)

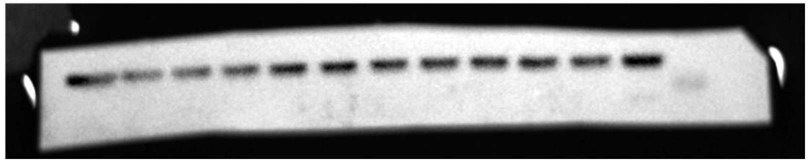

Gef+Lova+SQ p-AKT(473)

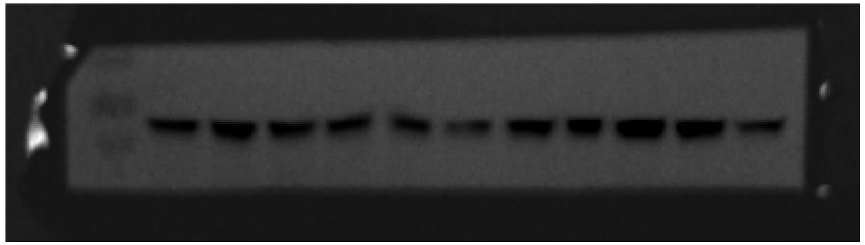

Gef+Lova+SQ AKT

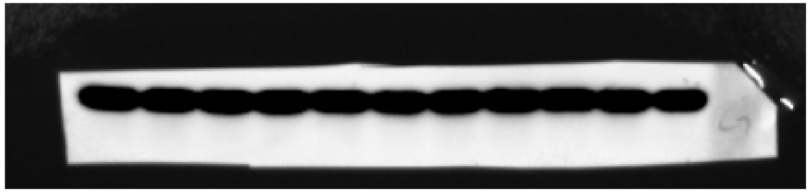

Gef+Lova+SQ GAPDH

Figure 4L

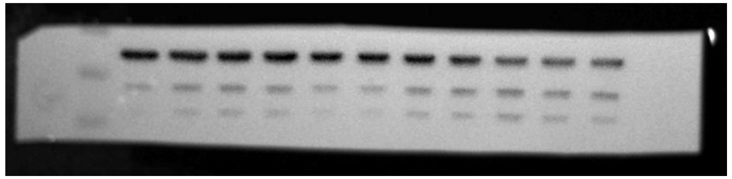

Gef+Lova+chol p-AKT(308)

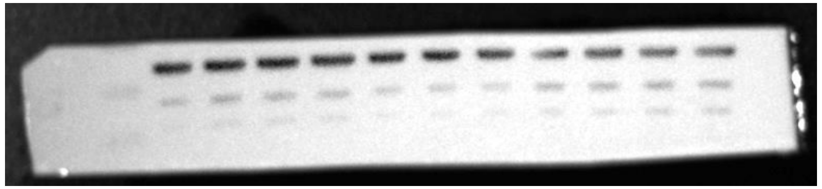

Gef+Lova+chol p-AKT(473)

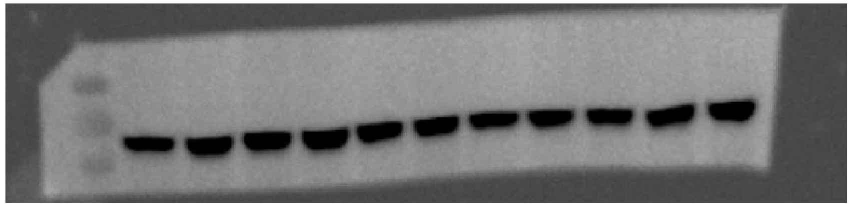

Gef+Lova+chol AKT

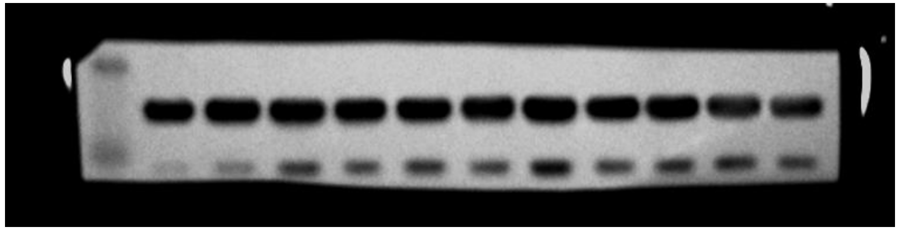

Gef+Lova+chol GAPDH

Figure 5A

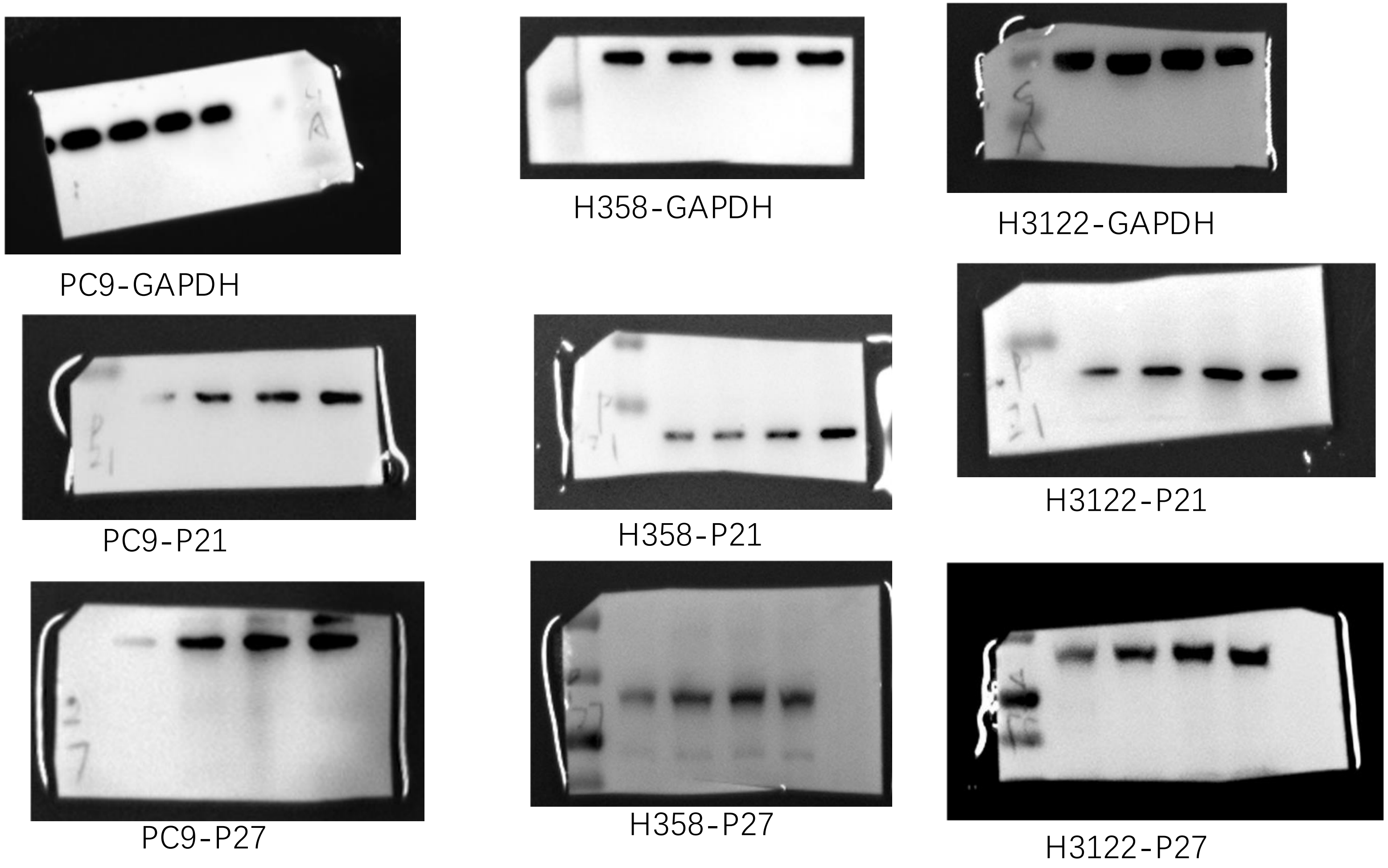
PC9-GAPDH
H358-GAPDH
H3122-GAPDH
PC9-P21
H358-P21
H3122-P21
PC9-P27
H358-P27
H3122-P27

Figure 6D

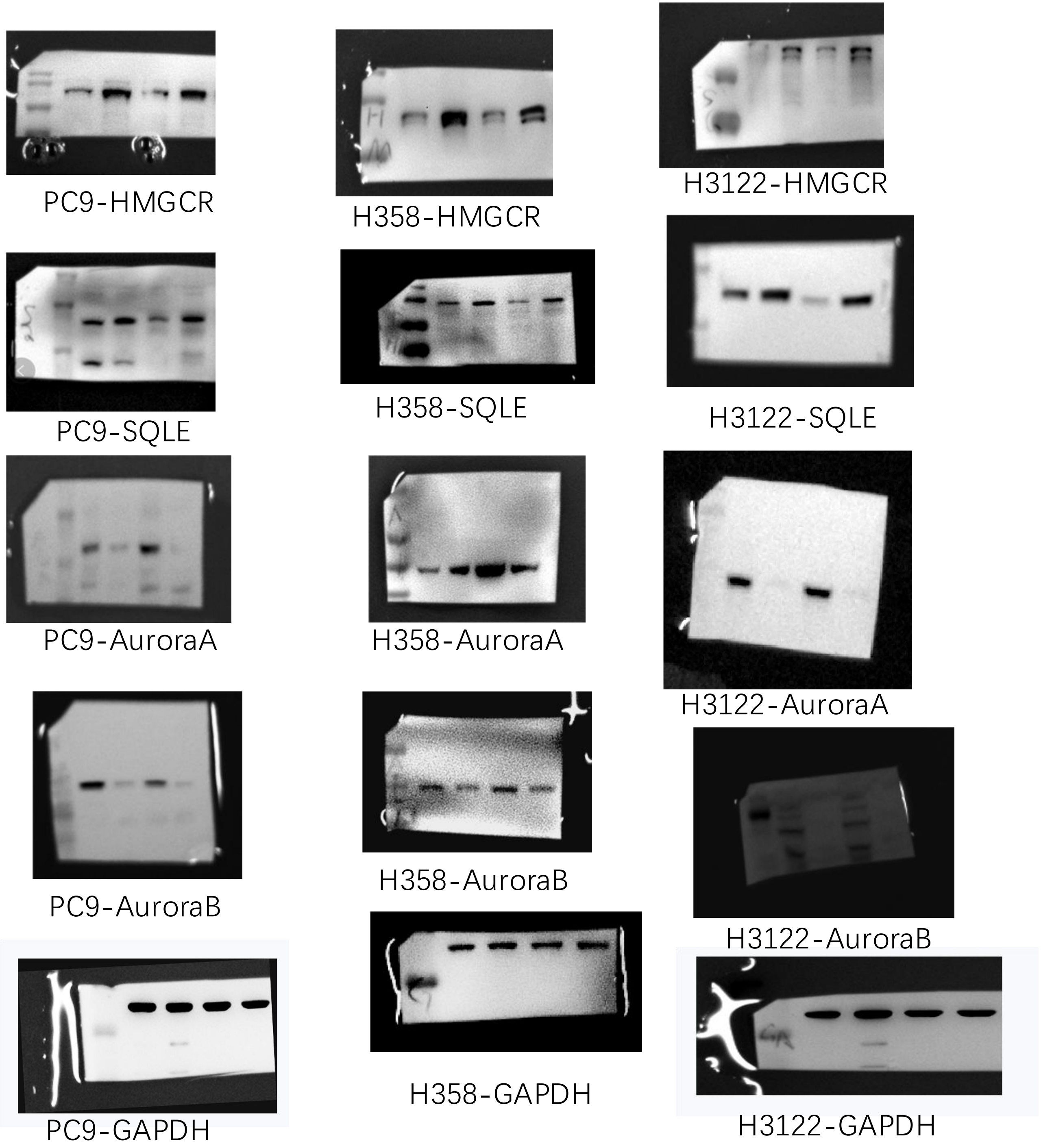
PC9-HMGCR
H358-HMGCR
H3122-HMGCR
PC9-SQLE
H358-SQLE
H3122-SQLE
PC9-AuroraA
H358-AuroraA
H3122-AuroraA
PC9-AuroraB
H358-AuroraB
H3122-AuroraB
PC9-GAPDH
H358-GAPDH
H3122-GAPDH

Supplementary figure 2D

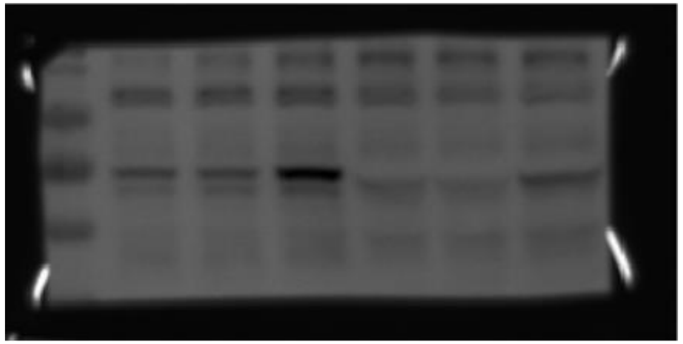

PC9-SREBP2

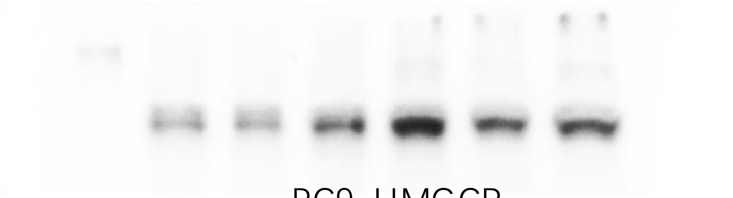

PC9-HMGCR

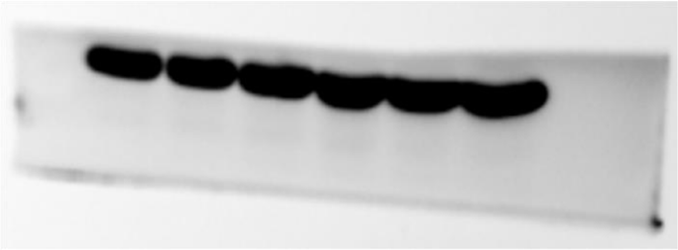

PC9-GAPDH

Supplementary figure 3A

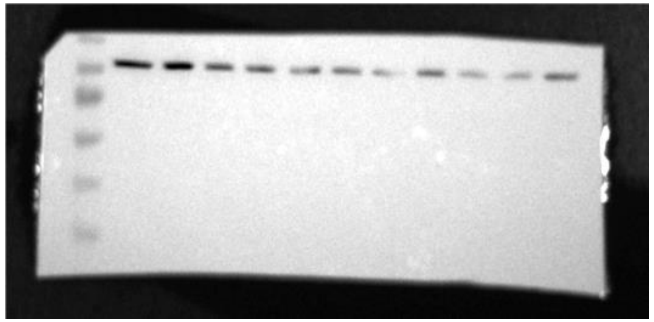

PC9-ATF6

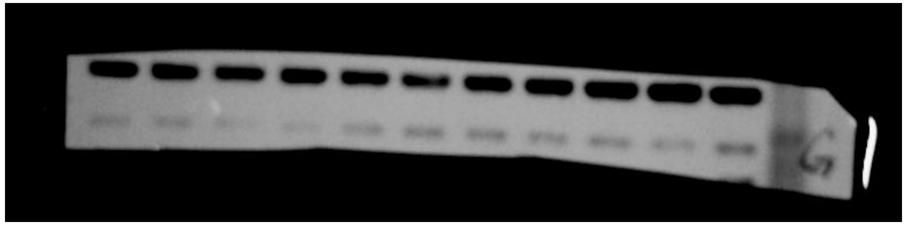

PC9-GAPDH

Supplementary figure 4 A

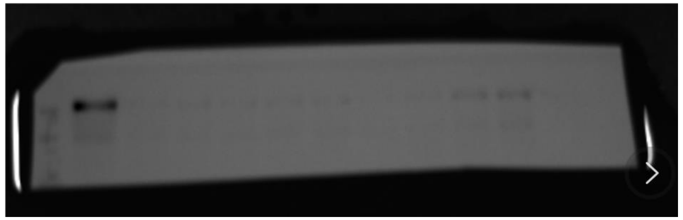

PC9-p-EGFR

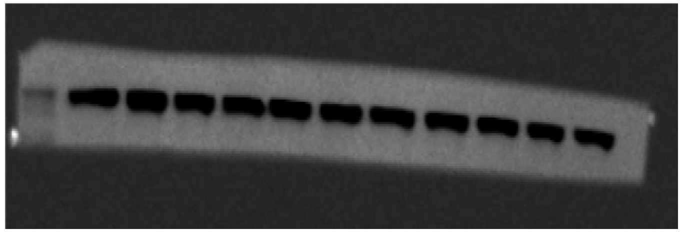

PC9-EGFR

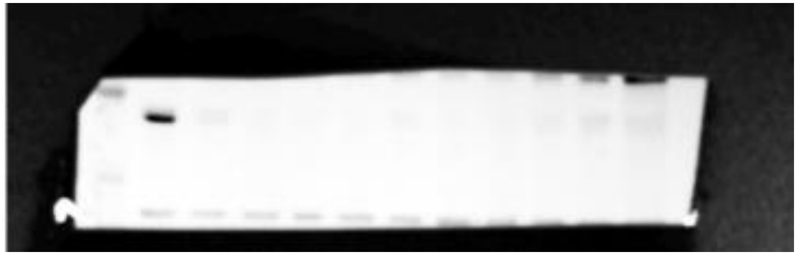

PC9-p-S6

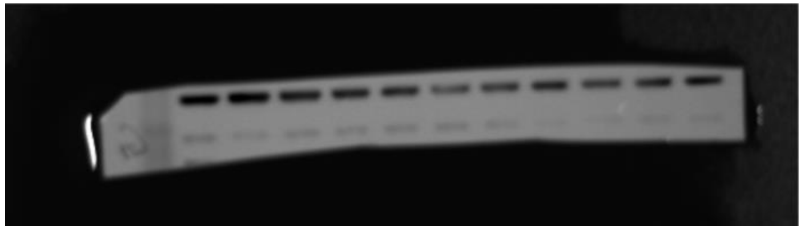

PC9-GAPDH

Supplementary figure 4 G

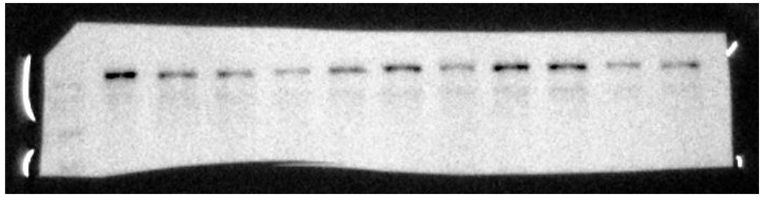

PC9-Ge+SQLE-p-EGFR

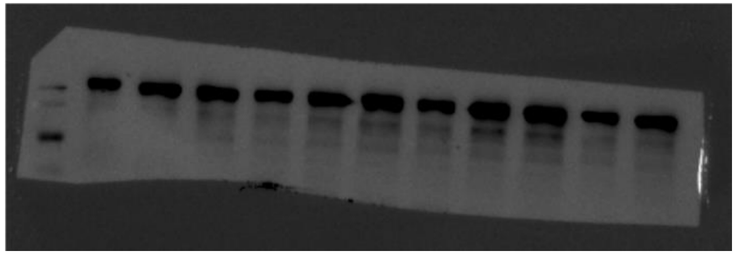

PC9-Ge+SQLE-EGFR

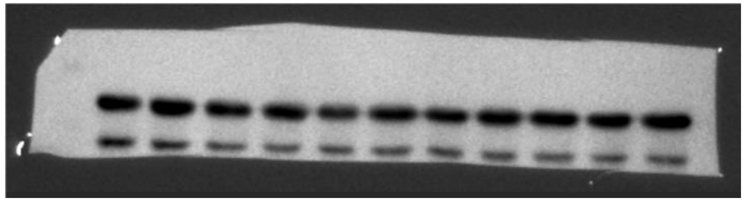

PC9-Ge+SQLE-GAPDH

Supplementary figure 4 I

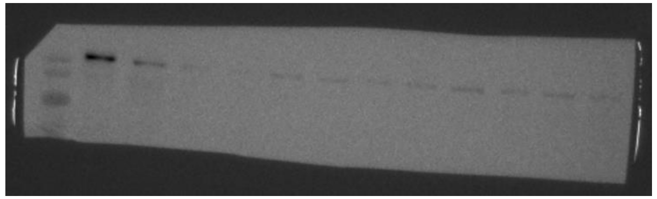

PC9-Ge+Cholesterol-p-EGFR

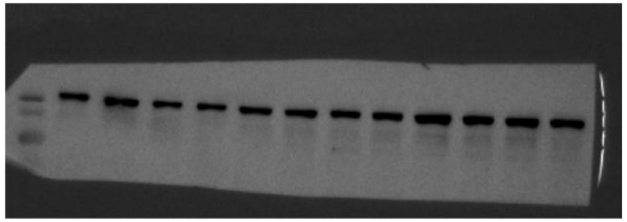

PC9-Ge+Cholesterol-EGFR

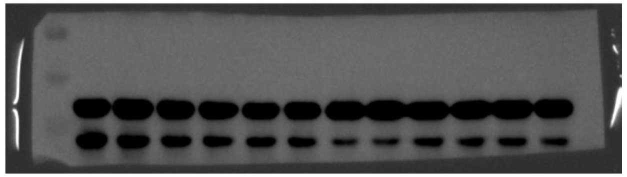

PC9-Ge+Cholesterol-GAPDH
